# Enhanced through‐bond energy transfer‐based bioorthogonal probe enables catalytic‐amplified and sensitive detection of microRNA‐21 for clinical lung cancer diagnosis

**DOI:** 10.1002/smo2.70067

**Published:** 2026-06-08

**Authors:** Yikun Li, Fuping Han, Haiqiao Huang, Danhong Zhou, Saran Long, Wen Sun, Jianjun Du, Jiangli Fan, Jing Ning, Chong Peng, Xiaojun Peng

**Affiliations:** ^1^ State Key Laboratory of Fine Chemicals Frontiers Science Center for Smart Materials Dalian University of Technology Dalian China; ^2^ College of Materials Science and Engineering Shenzhen University Shenzhen China; ^3^ Ningbo Institute of Dalian University of Technology Dalian University of Technology Ningbo China; ^4^ Pancreatic Disease Center Cancer Hospital of Dalian University of Technology Liaoning Cancer Hospital & Institute Shenyang China

**Keywords:** bioorthogonal chemistry, cyanine dye, enhanced TBET, lung cancer, miR‐21

## Abstract

The dysregulation of microRNA (miRNA) expression is closely linked to the pathogenesis of lung cancer, rendering the quantification of trace miRNAs clinically indispensable. However, achieving ultrasensitive detection of miRNAs in complex biological matrices, including living cells, tissues, and blood serum, remains significant challenges. To address this, we developed a fluorogenic detection platform for miR‐21 based on tetrazine‐mediated transfer (TMT) reactions. While TMT strategies have been previously explored, we report a refined probe design optimized through computational screening. By minimizing the energy gap (Δ*E*) between the fluorophore's emissive state and the tetrazine's dark state, we achieved an efficient quenching mechanism with a low background (fluorescence quantum yield <0.01). By integrating hybridization‐mediated target recycling with the inverse electron demand Diels‐Alder reaction, the Inverse Diels‐Alder Cycloaddition Reaction (IDCR) probe achieves signal amplification. Although the theoretical catalytic turnover is significantly enhanced at low target concentrations, the practical detection limit is primarily governed by the high signal‐to‐background ratio afforded by our optimized probe. This mechanism yields an ultralow limit of detection (3.58 × 10^−18^ M) with a broad linear range (100 aM to 100 nM). Building upon foundational bioorthogonal chemistry, the IDCR probe enables high‐contrast imaging in living cells and tissues and distinguishes lung cancer patients from healthy individuals.

## Graphical abstract

Inverse Diels‐Alder Cycloaddition Reaction was developed for ultrasensitive miR‐21 detection. Based on the enhanced Through‐Bond Energy Transfer and catalytic amplification, it shows a detection limit of 3.58 × 10^−18^ M and 100 aM–100 nM linear range. It enables high‐contrast cell/tissue imaging and distinguishes lung cancer patients in serum, promising non‐invasive diagnosis.

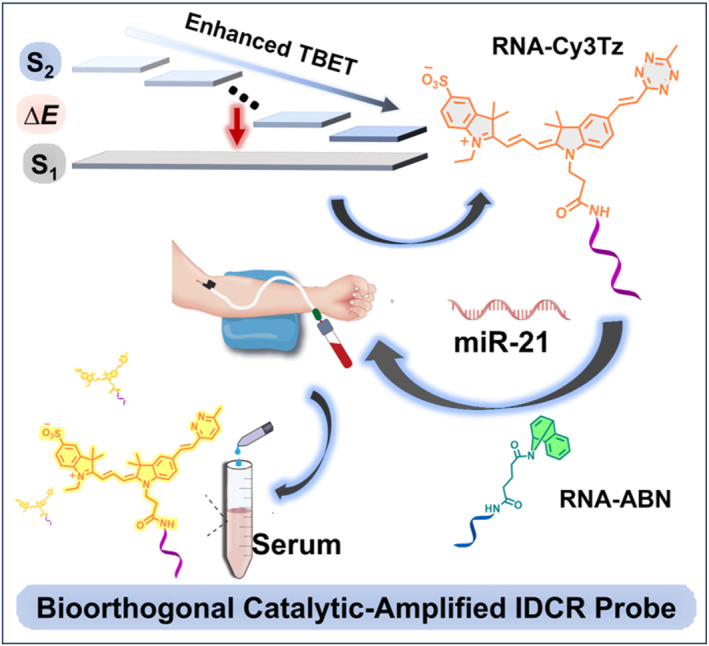

## 1 INTRODUCTION

Lung cancer remains the leading cause of cancer‐related mortality worldwide,[^1^, ^2^] primarily due to its asymptomatic nature in early stages.^[3]^ Consequently, there is an urgent need for highly sensitive and specific molecular diagnostic strategies capable of detecting the disease in its most treatable phase.[^4^, ^5^] MicroRNAs (miRNAs), which are short non‐coding RNAs, have emerged as promising biomarkers for cancer diagnosis, prognosis, and therapeutic monitoring.[^6^, ^7^, ^8^] Especially, their exceptional stability in blood and other bodily fluids makes them particularly suitable for noninvasive liquid biopsies.^[9]^ However, accurate quantification of circulating miRNAs remains a challenge[^10^, ^11^] due to their inherently low abundance at femtomolar in body fluids.[^12^, ^13^] Conventional detection methods, such as Quantitative Reverse Transcription Polymerase Chain Reaction (qRT‐PCR), northern blotting, and microarray analysis, often require complex sample processing, rely on sophisticated instruments, and lack the capability for direct and sensitive detection in serum or in situ imaging of dynamic miRNA changes within living cells and tissues.[^4^, ^14^] Therefore, achieving attomolar (aM) sensitivity, which surpasses conventional pico‐to‐femtomolar limits, is critical for advancing the early diagnostics of lung cancer.

To meet the requirements for sensitivity and specificity, strategies combining template‐triggered fluorescence with signal amplification capabilities have proven highly effective.[^15^, ^16^, ^17^] For successful practical application of this technique, the selection of reactive groups with high fluorogenic conversion efficiency that resist degradation and avoid cross‐reaction with biologically active groups is essential, along with sufficient reaction cycles per template to achieve an ideal detection limit.^[18]^ The inverse electron‐demand Diels‐Alder (IEDDA) reaction between tetrazines and strained dienophiles has recently gained attention for biomolecular detection due to its fast kinetics, excellent selectivity, and biocompatibility. [^19^, ^20^, ^21^, ^22^, ^23^, ^24^] For example, Devaraj et al. developed a tetrazine‐mediated transfer (TMT) reaction involving 7‐azabenzonorbornadiene derivatives (ABN) and fluorogenic tetrazines, realizing miRNA detection limits of approximately 5 pM, a pioneering work in nucleic acid‐templated tetrazine‐based detection.[^25^, ^26^] Another strategy demonstrates the coupling of tetrazine‐mediated bioorthogonal decaging with DNA cascade circuits for proximity‐driven sensing, with a reported detection limit of 2.1 μM.^[27]^ However, this reported methods' sensitivity is insufficient for reliable quantification of fM level miRNA in clinical media, highlighting the need for further optimization.

Mechanistically, the fluorescence quenching of fluorophores covalently linked to tetrazines relies on Through‐Bond Energy Transfer (TBET).[^28^, ^29^, ^30^] While TBET‐based tetrazine‐quenched dyes have been previously reported, their efficiency is highly sensitive to the linker architecture.[^31^, ^32^, ^33^] The tetrazine moiety possesses an excited state lower in energy than the emissive state of the chromophore, functioning as a dark state characterized by *n* → π* transitions. Upon photoexcitation, energy is transferred from the high‐energy emissive state (S_2_ or higher) of the chromophore to the low‐energy state (S_1_) of the tetrazine, thereby inhibiting fluorescence. However, the TBET efficiency varies significantly with the chromophore scaffold, linker type, and conjugation site. Inefficient TBET allows a fraction of the excitation energy to return to the ground state via radiative decay, resulting in high background fluorescence that compromises the sensitivity. Drawing inspiration from “enhanced Photoinduced Electron Transfer (PET)” strategies that successfully improved sensitivity for HClO detection by minimizing background noise in our former works,^[34]^ it is hypothesized that optimizing the tetrazine‐probe architecture to establish an “enhanced TBET” mechanism could yield superior performance of sensitivity. As far as we know, this “enhanced TBET” strategy—focused on minimizing the energy gap (Δ*E*) between the fluorophore's emissive state and the tetrazine's dark state—remains underexplored in the context of nucleic acid detection.

Herein, using a cyanine dye platform, we established an “enhanced TBET” mechanism in a tetrazine‐based Diels‐Alder system. To develop an efficient biorthogonal fluorescence sensing platform, screening numerous molecules to identify effective candidates is necessary. However, the traditional trial and error strategy demands substantial human resources and time for the synthesis of tetrazine derivatives. Therefore, intelligent molecular engineering was introduced to integrate molecular design, computational screening, and experimental validation of top‐ranked molecules.[^35^, ^36^, ^37^] By systematically investigating the structural and photophysical properties of 11 cyanine‐tetrazine conjugates in the literature,[^25^, ^38^, ^39^, ^40^] the adiabatic energy difference (Δ*E*) between the emissive and dark states was identified as the critical descriptor for predicting TBET efficiency through employing quantum chemical calculations. Guided by this principle, the engineered probe, Cy3Tz, exhibits enhanced TBET efficiency with a pre‐activation fluorescence quantum yield (QY) of less than 0.01. Furthermore, by integrating hybridization‐mediated target recycling mechanisms with the IEDDA reaction, we achieved enzyme‐free signal amplification, pushing the detection limit to the attomolar level. This work aims to address the sensitivity limitations of tetrazine‐based miRNA detection methods and provide a generalizable molecular design strategy for low‐background, high‐sensitivity bioorthogonal probes.

## 2 RESULTS AND DISCUSSION

### 2.1 Molecular computational screening and synthesis

**FIGURE 1:**
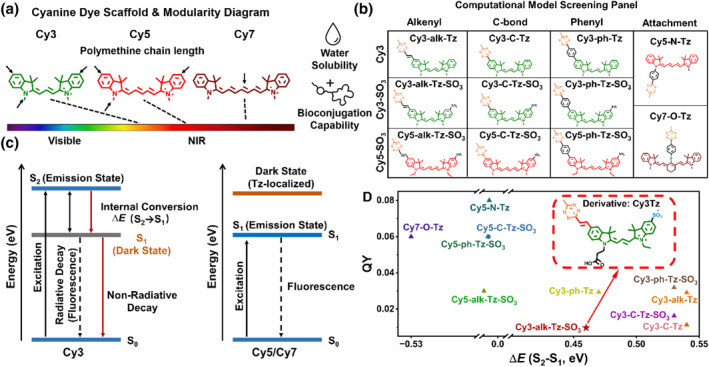
Schematic illustration of molecular computational screening. (a) Cyanine dye scaffold and modularity of Cy3, Cy5, and Cy7. (b) Computational model screening panel of cyanine‐tetrazine (Tz) derivatives. (c) Jablonski diagram illustrating the photophysical processes of the molecules (left: Cy3, right: Cy5/Cy7). (d) Correlation of S_2_ – S_1_ gap (Δ*E*) and quantum yield using ωB97X‐D/def2‐SVP.

The rational construction of high‐performance bioorthogonal turn‐on probes relies on the selection of the fluorophore.^[41]^ In this study, the cyanine dye family (Cy3, Cy5, and Cy7) was employed as the platform candidate, owing to its their exceptional structural modularity that permits systematic wavelength tuning across the visible to near‐infrared spectrum.^[42]^ This modularity facilitates fine‐tuning photophysical properties via adjusting the polymethine chain lengths and substituents, thereby optimizing conjugated system and molecular energy levels (Figure 1a). To systematically elucidate the impact of conjugation sites and fluorophore categories on quenching and activation performance, a comprehensive computational model was developed encompassing eleven tetrazine‐cyanine conjugates reported in the literature (Figure 1b).[^25^, ^28^, ^38^, ^39^, ^40^, ^43^] To govern the general structure‐property relationships of probes, specifically examining variations in fluorophore cores (Cy3/Cy5/Cy7), linker chemistry (alkenyl, phenyl, C‐bond, ether), and attachment sites (indole ring vs. N‐atom) were optimized for maximizing quenching efficiency and minimizing background fluorescence.

Density functional theory (DFT) was employed to optimize the ground‐state geometries and obtain vertical excited‐state structures at the ωB97X‐D/Def2‐SVP level (water/Solvation Model based on Density solvation model).[^44^, ^45^, ^46^] Analysis of the orbital energy levels (Table S1) reveals that the bright states of all probes correspond to the π → π* (Highest Occupied Molecular Orbital [HOMO] → Lowest Unoccupied Molecular Orbital [LUMO]) transition, while the dark states correspond to the *n* → π* (HOMO‐2 → LUMO + 1) transition. The Cy3 series exhibits a significantly smaller energy gap (Δ*E*) between the LUMO (bright) and LUMO + 1 (dark) states compared to Cy5 and Cy7, predicting a stronger electron transition capability. Furthermore, orbital overlap (Sr) analysis between these states shows a clear positive correlation with quenching efficiency: higher overlap yields more pronounced TBET and lower fluorescence QY, whereas spatial distance (D) lacks a linear relationship. Structurally, linking the tetrazine to Cy3 via a C=C bond maximizes this orbital overlap, providing enhanced TBET potential.

Time‐Dependent Density Functional Theory calculations provided deeper mechanistic insights.[^47^, ^48^] Vertical excitation and hole‐electron analyses of the optimized ground‐state (S_0_) geometries (Table S2) reveal that for long‐wavelength dyes (Cy5 and Cy7), the tetrazine‐localized “dark state” lies above the emissive state, rendering the S_1_ state emissive. This alignment is thermodynamically unfavorable for the TBET, consistent with the high background fluorescence (QY >3%). Conversely, for the Cy3 series, the emissive state is the higher‐energy S_2_ state (characterized by large oscillator strength), while S_1_ state functions as the dark state. Upon excitation, competitive relaxation occurs: radiative decay (fluorescence) versus internal conversion to the S_1_ (non‐radiative decay). Enhancing this internal conversion is critical for effective fluorescence quenching. These specific state assignments were also robustly validated using the M06‐2X/def2‐SVP function (Figures S5 and S6).

Further analysis identifies the adiabatic energy difference (Δ*E*) between the S_2_ and S_1_ states as a critical thermodynamic descriptor for predicting TBET efficiency. As shown in Figure 1c,d, a strong correlation was observed: while a negative Δ*E* hinders TBET, a positive Δ*E* with a small absolute value significantly facilitates the S_2_ → S_1_ internal conversion, thereby maximizing the TBET efficiency. Applying this descriptor to the structural screening, Cy3‐alk‐Tz‐SO_3_ was identified as possessing the minimal calculated gap (Δ*E* = 0.2998 eV), which corresponds perfectly with its experimentally determined lowest fluorescence QY (0.0097).^[38]^ This high correlation validates the strategy of connecting tetrazine to the Cy3 terminus via a double bond to construct “enhanced TBET” probes.

This mechanism represents a sophisticated extension, rather than a contradiction, of the “introduction of low‐energy dark states” concept. It elucidates the pivotal role of the conjugated double bond (alkenyl linker) in mediating efficient TBET. Furthermore, the incorporation of the ‐SO_3_ group effectively narrows the adiabatic energy difference (Δ*E*) between the S_2_ and S_1_ states, thereby thermodynamically favoring internal conversion and amplifying the quenching efficiency. In summary, the convergence of computational and experimental data establishes minimizing Δ*E* as a core design principle and confirms the Cy3‐alk‐Tz‐SO_3_ architecture as the optimal configuration for achieving superior signal‐to‐background ratios.^[20]^

**SCHEME 1:**
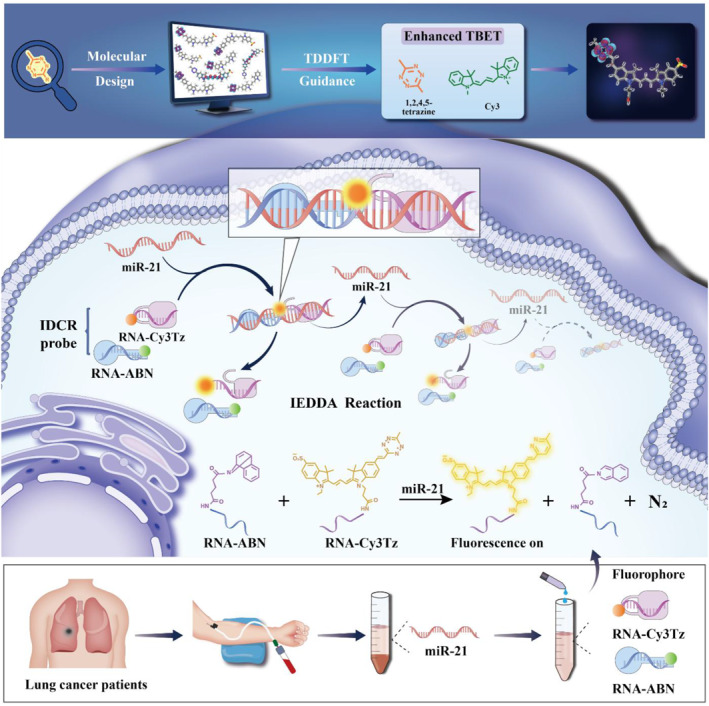
Schematic illustration of the catalytic‐amplified detection of microRNA‐21 using an enhanced Through‐Bond Energy Transfer‐based bioorthogonal Inverse Diels‐Alder Cycloaddition Reaction probe for lung cancer diagnosis.

Based on the computational screening results, a miRNA detection probe was designed. The optimal derivative Cy3Tz, modified with a carboxyl group for RNA conjugation, was synthesized as the key structure (Figures S1, S2 and S14–S22), and its photophysical properties were thoroughly characterized to confirm efficient fluorescence quenching by the tetrazine moiety. UV‐vis absorption and fluorescence spectra of both Cy3 and Cy3Tz were acquired in various solvents (Figure S5). Upon conjugation with tetrazine, the absorption maximum exhibited a bathochromic shift of approximately 20 nm, indicating extended conjugation and altered electronic properties. More importantly, significant fluorescence quenching was observed across all solvents tested, with an average quenching efficiency of ∼88%, confirming the effective suppression of Cy3 emission by the tetrazine group. This validated Cy3Tz effectively suppressed initial fluorescence. To leverage this quenched probe in a bioorthogonal context, we further employed Cy3Tz in templated tetrazine reactions using ABN as strained dienophiles, which undergo efficient TMT (Scheme 1).

### 2.2 Design and synthesis of the final RNA‐conjugated IDCR‐probe

To computationally model the reaction feasibility, we investigated the Diels‐Alder cycloaddition between Cy3Tz and ABN using DFT at the B3LYP/6‐31G* level, a well‐established method for balancing accuracy and computational efficiency. Geometry optimization of the reactants and their intermediate complex revealed a key geometric parameter: the distance between the terminal nitrogen atoms of the connecting bases in the optimized complex was 1.4 nm (Figure S10a). This result implies a critical proximity criterion; separations shorter than this value likely permit the necessary orbital overlap for a viable cycloaddition, underscoring the importance of molecular pre‐organization in this reaction.

Building upon the validated Cy3Tz module, Cy3Tz was linked to the strands of 5′‐amine‐modified oligonucleotides, while ABN/Bicyclo [6.1.0] nonyne (BCN) was linked to the strands of 3′‐amine‐modified oligonucleotides, forming the IDCR‐probe composed of RNA‐Cy3Tz and RNA‐ABN for specific miRNA detection. For comparison, a control probe consisting of RNA‐Cy3Tz and RNA‐BCN was also prepared.

The operational principle of Inverse Diels‐Alder Cycloaddition Reaction (IDCR) is illustrated in Scheme 1 and Figure S3. When two complementary antisense probes, one bearing Cy3Tz and the other bearing the dienophile (ABN), hybridize adjacently to a target RNA sequence, the tetrazine and dienophile are brought into close proximity (<1.4 nm as per DFT analysis). This spatial confinement enables a highly efficient IEDDA cycloaddition. The reaction reduces the tetrazine to a dihydropyridazine derivative, which abolishes its quenching effect and restores Cy3 fluorescence. Crucially, the initial cycloaddition is followed by a spontaneous retro‐Diels‐Alder reaction that fragments the adduct.^[49]^ Because this TMT process transfers the functional group rather than ligating the probes, it prevents the formation of a tightly bound extended duplex. The resulting short fluorescent product readily dissociates via dynamic strand exchange at 37°C, continuously freeing the template to recruit fresh probes for multiple turnover cycles and enzyme‐free signal amplification. All probe sequences used for targeting different miRNAs are detailed in the Supporting Information.

### 2.3 Feasibility of IDCR‐probe for microRNA detection in vitro

**FIGURE 2:**
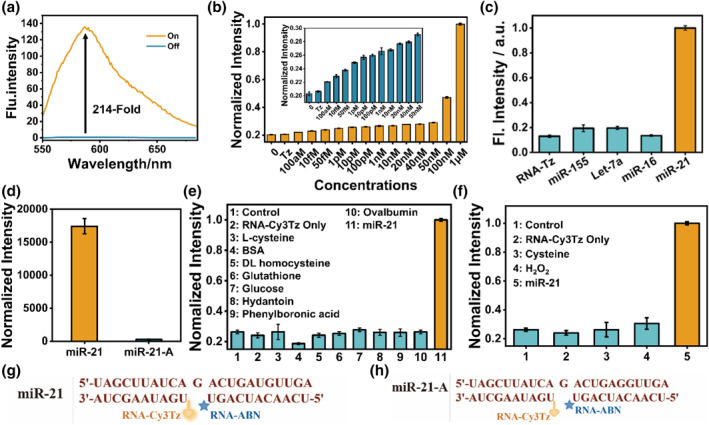
Feasibility, specificity and stability of IDCR‐probe for microRNA detection in vitro. (a) Fluorescence turn‐on of the IDCR probe with/without addition of target miR‐21. (b) Fluorescence intensity determination at various RNA template concentrations from 100 aM to 1 μM, with an expanded view of the 0–50 nM range shown in the inset. (c) Specificity of the IDCR probe against various highly abundant serum miRNAs. (d) Distinguishing single‐mismatch wrong probes from perfect match IDCR probes. (e) Fluorescence responses of RNA‐Cy3Tz and RNA‐ABN (1 μM) toward various analytes; 50 nM RNA template and 5 mM (105 equiv) all other analytes. (f) Normalized fluorescence emission signal of 1 μM of IDCR probes after 2 h of incubation with different reactive additives showing the stability of the probes with respect to the reactive additives. Sequences for (g) miR‐21 and (h) miR‐21‐A templates. Error bars show the standard deviations of three replicates. *λ*_ex_ = 520 nm, *λ*_em_ = 587 nm. IDCR, Inverse Diels‐Alder Cycloaddition Reaction.

miR‐21 was selected as the model target in the fluorescence‐based detection, as it is a well‐established oncomiR notably upregulated in lung cancer.^[50]^ To confirm the template‐dependent nature of the fluorescence turn‐on, control experiments were first performed in the absence of the target template. When only the two probe components (RNA‐Cy3Tz and RNA‐ABN) were mixed in solution, fluorescence increased by 0.3% after 2 h of incubation at 37°C (Figure S9b). In contrast, adding the template miRNA under identical physiological conditions (37°C for 2 h) led to a 214‐fold restoration of the fluorescence signal (Figure 2a), demonstrating the high stability of the system and its suitability for practical biological applications.

Subsequently, the response of the IDCR system was evaluated using a synthetic miR‐21 template under 37°C for 2 h. The concentrations of RNA‐Cy3Tz and RNA‐ABN were fixed at 1 μM, and the fluorescence intensity was normalized to that of the reaction containing 1 μM RNA template. The target concentration was then varied from 100 aM to 1 μM, with control groups including a no‐template condition and a Tz‐only group (RNA‐Cy3Tz alone). As shown in Figures 2b and S9c, the fluorescence intensity increased progressively with higher target concentrations, indicating a dose‐dependent activation of the probe via the template‐promoted IEDDA reaction. Furthermore, a linear relationship was observed between the normalized fluorescence intensity and the logarithm of the miRNA concentration (log C), as illustrated in Figure S10c. Based on this calibration, the limit of detection for miR‐21 was determined to be 3.58 × 10^−18^ M, highlighting the exceptional sensitivity of the IEDDA‐based IDCR system. Notably, in Figure S9d, when RNA‐Cy3Tz and RNA‐BCN were paired for detection, the fluorescence recovery was observed to be lower than that achieved with the IDCR system under identical conditions, which can be attributed to the comparatively slower reaction kinetics between BCN and tetrazine. Thus, the IDCR probe constitutes a versatile and programmable platform for miRNA detection, achieving a detection limit of 3.58 aM that is competitive with the most sensitive fluorescent methods reported (Table S4).

Interestingly, the exceptional sensitivity of the IDCR probe stems from the synergy between its efficient catalytic turnover and the “Enhanced TBET” mechanism (Table S3). By calculating the apparent turnover number in the linear response range, it was confirmed that the target miRNA acts as a catalyst in multiple reaction cycles. Also, at lower target concentrations, a greater proportion of unreacted probes remains available for successive cycles of hybridization and reaction, leading to signal amplification. This catalytic target recycling works in tandem with the “Enhanced TBET” mechanism to achieve the attomolar sensitivity.

### 2.4 Specificity, selectivity and stability of IDCR‐probe for microRNA detection in vitro

Based on the understanding that miRNA family members can differ by as little as a single nucleotide,^[15]^ we next evaluated the specificity of our IDCR probes against a series of endogenous miRNA‐like sequences. To challenge the system's selectivity, we synthesized a mismatched RNA sequence (miR‐21‐A, Figure 2h) containing a single‐base mismatch (at position 10) relative to the fully complementary miR‐21 target. Additionally, to account for potential interference in biological samples, we tested the probe against several other abundant serum miRNAs, including miR‐155, miR‐16 and let‐7a (Figure 2c). In the specificity assays, a fixed concentration (100 nM) of the complementary template was used, with the probe components (RNA‐Cy3Tz and RNA‐ABN) each maintained at 1 μM. As shown in Figure 2c,d, the system demonstrated excellent discrimination, with a strongly significant fluorescence signal observed for the perfectly matched target compared to the mismatched probes (*P* < 0.0001), underscoring the general robustness of the IEDDA cycloaddition for specific molecular recognition.

To assess the potential interference from endogenous biomolecules on miRNA detection, we introduced the IDCR probes (RNA‐Cy3Tz and RNA‐ABN, 1 μM each) into a phosphate buffer saline (PBS) buffer system containing various biologically relevant compounds, including L‐cysteine, DL‐homocysteine, glutathione, glucose, hydantoin, phenylboronic acid, bovine serum albumin, and ovalbumin. The fluorescence intensity was normalized to a control group containing only IDCR in PBS. As shown in Figure 2e, a significant increase in fluorescence emission (approximately 5‐fold) was observed exclusively in the presence of the target miR‐21 template, whereas all other additives induced only minor signal fluctuations with changes remaining within ±0.05 of the baseline level over the 2‐h incubation period.

We further evaluated the chemical stability of the IDCR probes under potentially challenging conditions. The probes were incubated for 2 h with a large excess (5 mM, 105 equivalents) of reactive species, such as the oxidant hydrogen peroxide (H_2_O_2_) or the thiol nucleophile cysteine—the background fluorescence remained low (<0.15 a.u.). Subsequent addition of a sub‐stoichiometric amount of the miR‐21 template (100 nM, 10% equiv) triggered a fluorogenic response (Figure 2f), yielding about a 5‐fold fluorescence recovery. These findings collectively demonstrate that the IDCR system possesses excellent anti‐interference capability and robust chemical stability against various bioactive compounds, highlighting its potential for reliable operation in complex biological environments.

### 2.5 MicroRNA imaging in living cells

Following the successful in vitro validation of the IDCR probe for miR‐21 detection, we proceeded to evaluate its performance for imaging endogenous miRNA in living cells. The oncomiR miR‐21 is known to be overexpressed in a range of cancers.^[51]^ Accordingly, we selected three relevant cancer cell lines: pancreatic cancer (PANC‐1), lung adenocarcinoma (A549), and breast cancer (MCF‐7). Prior to imaging, Methylthiazolyldiphenyl‐tetrazolium bromide experiments were carried out to assess cytotoxicity.^[52]^ The results confirmed that the IDCR‐probe exhibited low cytotoxicity, with cell viability remaining between 100% and 109% at a low concentration (0.625 μM) and 88%–95% even at a high concentration (30 μM) after 24 h of incubation (Figure S11).

**FIGURE 3:**
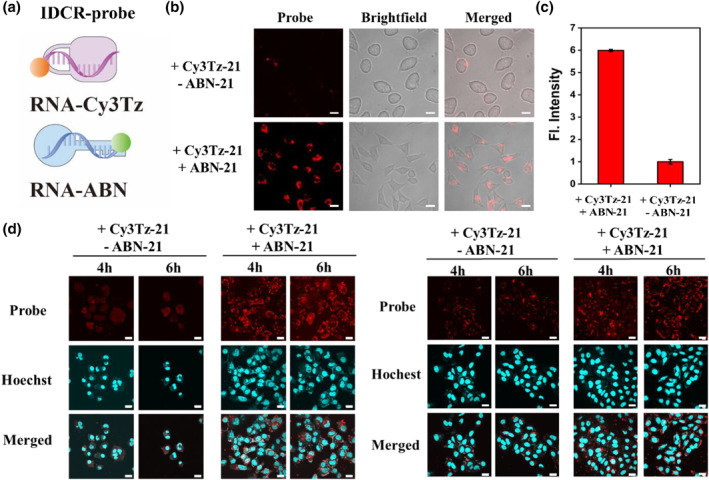
Confocal fluorescence imaging in living cells. (a) Diagram of IDCR‐probe. (b) Confocal fluorescence imaging of miR‐21 in MCF‐7 cells incubated with RNA‐Cy3Tz and RNA‐ABN. (c) Average fluorescence intensity obtained from fluorescence images after 6 h of incubation in MCF‐7 cells with and without RNA‐ABN. (d) Confocal fluorescence imaging of miR‐21 in PANC‐1 and A549 cells after incubation with Hoechst 33342 and IDCR probes for 4 and 6 h. Scale bar = 20 μm. IDCR, Inverse Diels‐Alder Cycloaddition Reaction.

For live‐cell imaging, the cells were transfected with a mixture of RNA‐Cy3Tz (2 μg) and RNA‐ABN (2 μg) using Lipofectamine 3000 in Opti‐MEM (a schematic of the procedure is shown in Figure 3a). First, to determine the optimal incubation time for signal development, we monitored the fluorescence in PANC‐1, MCF‐7, and A549 cells over a period of 0–6 h (Figure 3b–d). A robust fluorescence signal (approximately 6‐fold) was observed in all cell lines by 6 h, with no significant further increase upon longer incubation, establishing 6 h as the optimal time point for subsequent experiments. This robust staining confirmed the effective detection of endogenous miR‐21 across all three cancer cell lines. In contrast, control experiments omitting the RNA‐ABN component showed markedly reduced fluorescence. This visual contrast was quantitatively validated in MCF‐7 cells, where the average fluorescence intensity measured ∼6‐fold higher in the presence of IDCR probe compared to the RNA‐ABN‐omitted control. Additionally, a control with RNA‐ABN alone yielded negligible background signal across all cell lines (Figure S12), confirming that the observed turn‐on fluorescence is specific to the IEDDA reaction between the two probe components in the presence of the target miRNA. Furthermore, time‐dependent imaging confirmed that the background originates from the intrinsic emission of the Cy3Tz rather than progressive biological degradation.

Moreover, to further validate the specificity and quantitative capability of the IDCR‐probe against endogenous miR‐21 expression levels, we extracted total RNA from five distinct cell lines (3T3, LO2, MCF‐7, A549, and PANC‐1). The extracted RNA samples were incubated with IDCR probe (RNA‐Cy3Tz and RNA‐ABN, 1 μM) for 2 h. As shown in Figure S13, the fluorescence intensity from MCF‐7, A549 and PANC‐1 cells was significantly higher (1.6–2.5‐fold) than that in normal cell lines (LO2, 3T3). This differential expression pattern aligns well with the expected miR‐21 overexpression in malignancies and is consistent with the trend obtained by qRT‐PCR analysis.

### 2.6 Confocal fluorescence imaging in tissues

**FIGURE 4:**
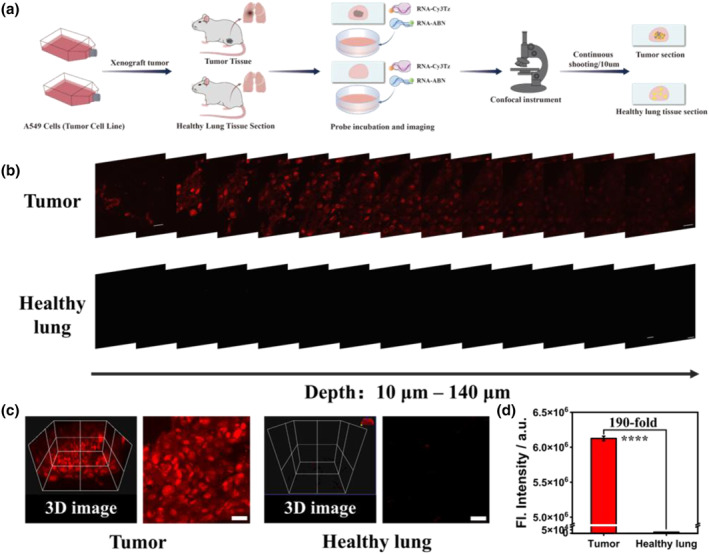
Confocal fluorescence imaging in tissues. (a) Operation schematic diagram of tissue imaging. Confocal fluorescence imaging of (b) subcutaneous lung tumor (up) and healthy lung's tissues (down) after incubation with Inverse Diels‐Alder Cycloaddition Reaction probe (RNA‐Cy3Tz and RNA‐ABN, 2 μg for 30 min, respectively). (c) Corresponding 3D images were accumulated along the *Z*‐direction at depths of 0–140 μm for tumor (left) and normal tissues (right). *λ*_ex_ = 488 nm, *λ*_em_ = 520−560 nm. (d) Quantitative analysis of fluorescence intensity of tumor and healthy lung issues. Scale bar = 20 μm.

Building upon the successful cellular imaging results, we sought to evaluate the potential of the IDCR‐probe for clinical translation by assessing its performance in more complex tissue environments. Effective penetration and labeling within tissues represent a significant challenge for many molecular probes due to hindered diffusion and non‐specific binding.^[53]^ To this end, we established a mouse model bearing subcutaneous tumors derived from A549 cells (human lung cancer) and prepared tissue sections from these tumors as well as from the lungs of healthy mice (Figure 4a).

The tissue sections were incubated with a solution of IDCR probe (RNA‐Cy3Tz and RNA‐ABN, 1 μM, respectively) for 30 min. Fluorescence confocal microscopy revealed that the IDCR‐probe achieved a notable penetration depth of approximately 140 μm within this short incubation period (Figure 4b–d), demonstrating its rapid diffusion and satisfactory permeability in both tumor and normal tissues. More importantly, the IDCR probe enabled clear discrimination between tumor and normal tissues; a significantly stronger fluorescence signal was observed in the tumor section compared to the normal lung tissue. Quantification of the fluorescence intensity in superimposed fields revealed that the tumor tissue exhibited a fluorescence signal approximately 190 times stronger than that of the normal lung tissue. This contrast is consistent with the elevated miR‐21 expression expected in malignant tissues and highlights the probe's ability to generate specific contrast in a complex tissue environment.

### 2.7 MicroRNA detection with IDCR‐probe and qRT‐PCR in clinical serum samples

**FIGURE 5:**
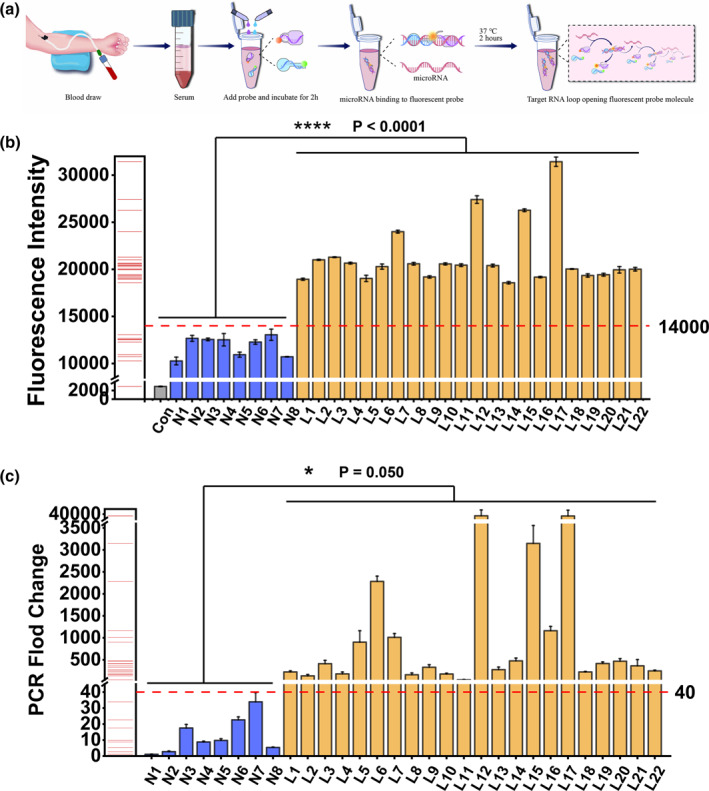
Target microRNA detection in serum samples. (a) Operation schematic diagram of microRNA detection in serum samples. Fluorescence responses of endogenous miR‐21 with each serum samples, including healthy people (N1 – N8) and lung cancer patients (L1 – L22), for 2 h under (b) Inverse Diels‐Alder Cycloaddition Reaction probe, compared with (c) qRT‐PCR strategy. Statistical significance was determined using a one‐tailed two‐sample *t*‐test. *p < 0.05, **p < 0.01, ***p < 0.001, ****p < 0.0001.

The outstanding performance of the IDCR platform in cellular and tissue imaging supports its potential for challenging bioanalytical diagnostics, such as the non‐invasive diagnosis of lung cancer. This disease is often diagnosed at advanced stages due to slow early growth, vague symptoms, and the lack of sensitive early screening tools. miR‐21 has been widely recognized as an oncogene that is upregulated in lung cancer and is associated with tumor progression and poor prognosis. However, accurately quantifying miRNA in serum remains difficult due to extremely low RNA content, minimal target abundance, inefficiencies in extraction, and high sample‐to‐sample variability—factors that often limit the discriminative power of conventional methods like qRT‐PCR (*P* = 0.050 in our comparative analysis, Figure 5c).

In contrast, the IDCR‐probe offers distinct advantages for direct serum‐based miRNA detection. As a homogeneous fluorescence assay, it operates on an “add‐and‐measure” basis, producing stable results within 10 min to 24 h (data shown for 2 h at 37°C) and eliminating the need for RNA extraction, thereby avoiding associated recovery bias (Figure 5a). Moreover, the system incorporates a catalytic turnover mechanism that provides signal amplification, enabling the sensitive detection of low‐abundance miRNAs without pre‐enrichment.

To assess clinical applicability, we performed a blinded test on 30 human serum samples collected through hospital collaboration (8 healthy donors and 22 lung cancer patients). As shown in Figure 5b, the IDCR‐probe successfully identified significantly elevated miR‐21 levels in cancer patients with high statistical confidence (*p* < 0.0001), and the results were fully consistent with clinical CT diagnoses, providing evidence for its reliability in clinical settings. This performance surpassed that of qRT‐PCR in terms of group discrimination and highlights the platform's strong potential for non‐invasive lung cancer testing. Notably, our platform also yielded a substantially larger effect size (Cohen's *d* = 4.98) compared to qPCR (Cohen's *d* = 0.71), indicating superior capability to differentiate between the tested groups.

## 3 CONCLUSION

In summary, we have developed the IDCR‐probe, a novel fluorescence sensing platform for the ultrasensitive detection of miRNA‐21. A key innovation of this work is the establishment and application of an “Enhanced TBET” mechanism, rationally guided by computational quantum chemical screening. This approach enabled the engineering of a cyanine fluorophore‐tetrazine conjugate (Cy3Tz) within an optimized RNA scaffold, achieving near‐complete fluorescence quenching (Φ < 1%) and a 214‐fold activation upon reaction. The platform operates via a proximity‐induced, IEDDA cycloaddition between the engineered RNA‐Cy3Tz and a dienophile‐tagged RNA (RNA‐ABN). By integrating this highly efficient fluorogenic reaction with a hybridization‐mediated target recycling strategy, the system achieves remarkable catalytic signal amplification. Working in tandem with the Enhanced TBET mechanism, the IDCR probe results in an exceptional detection limit of 3.58 × 10^−18^ M and a broad linear range across nine orders of magnitude. The IDCR‐probe demonstrates high specificity and enables wash‐free, high‐contrast imaging of endogenous miRNA‐21 in living cells and tissues, effectively distinguishing cancer cells from normal counterparts. Critically, its clinical utility was validated through the analysis of human serum samples, where it successfully discriminated between lung cancer patients and healthy individuals, highlighting its significant potential for non‐invasive diagnostics. This work not only presents a powerful and reliable tool for detecting nucleic acids in complex biological matrices but also provides a general design framework—combining computational prediction with mechanistic photophysical engineering—for developing advanced fluorescence probes. To fully realize the translational potential of this platform, future work will focus on expanding its multiplexed detection capabilities through the integration of logic‐gate designs or spatially encoded microarray formats.

## CONFLICT OF INTEREST STATEMENT

The authors declare no conflicts of interest.

## ETHICS STATEMENT

All animal procedures were performed in accordance with the guidelines for Care and Use of Laboratory Animals of Dalian Medical University and approved by the Dalian University of Technology Animal Care and Use Committee. Human participant experiments were performed with the approval of the Medical Ethics Committee of Liaoning Provincial Cancer Hospital (approval number: BQ20241205).

## Supporting information

Supporting Information S1

## Data Availability

The data that support the findings of this study are available from the corresponding author upon reasonable request.
