# Supplementary material for: Enhanced through‐bond energy transfer‐based bioorthogonal probe enables catalytic‐amplified and sensitive detection of microRNA‐21 for clinical lung cancer diagnosis
Source: Smart Mol. 2026 Jun 8:e70067. Online ahead of print. doi: 10.1002/smo2.70067 (PMC13399113; doi:10.1002/smo2.70067)
Supplement: Supplementary file 1 — Supporting Information S1 [file SMO2-9999-0-s001.docx]

Supporting Information

# Enhanced Through-Bond Energy Transfer-Based Bioorthogonal Probe Enables Catalytic-Amplified and Sensitive Detection of MicroRNA-21 for Clinical Lung Cancer Diagnosis

Yikun Li^1^, Fuping Han^1^, Haiqiao Huang^3^, Danhong Zhou^1^, Saran Long^1^, Wen Sun^1,4^, Jianjun Du^1,4*^, Jiangli Fan^1,4*^, Jing Ning^2*^, Chong Peng^1,4*^, Xiaojun Peng^1,3^

1 State Key Laboratory of Fine Chemicals, Frontiers Science Center for Smart Materials, Dalian University of Technology, Dalian 116024, China

2 Pancreatic Disease Center, Cancer Hospital of Dalian University of Technology, Liaoning Cancer Hospital & Institute, Shenyang 110042, China

^3^ College of Materials Science and Engineering, Shenzhen University, Shenzhen, 518060, China

^4^ Ningbo Institute of Dalian University of Technology, Dalian University of Technology, Ningbo 315016, China.

*Corresponding Authors: Jianjun Du: [dujj@dlut.edu.cn](mailto:dujj@dlut.edu.cn); Jiangli Fan: fanjl@dlut.edu.cn; Jing Ning: jning@cmu.edu.cn; Chong Peng: [pengchong@dlut.edu.cn](mailto:pengchong@dlut.edu.cn)

Experimental Methods

General information and materials

All reagents used were obtained from commercially available analytical reagents (A.R.) without further purification. Solvents used were purified via standard methods. Flash column chromatography was performed using silica gel (100–200 mesh) obtained from Qingdao Ocean Chemicals. The final product was purified by SepaBean^TM^ machine Flash Chromatography System from Santai Technologies Inc. and HPLC System from the Shimadzu Corporation. NMR spectra were recorded by using a Bruker Avance II instrument (400MHz). Mass spectrometric data were achieved with HP1100LC/MSD MS instruments. Double-distilled purified water used in all experiments was from Milli-Q systems. The spectra were performed on Agilent Cary 60 UV-Vis Spectrophotometer, Agilent CARY Eclipse fluorescence spectrophotometer, Thermo Scientific™ Varioskan™ LUX multimode microplate reader and Thermo Scientific NanoDrop™3300 Fluorospectrometer. Fluorescence images were obtained with a confocal laser scanning microscope (Olympus FV3000) DMEM was purchased from Tansoole Agent (China). PBS was purchased from Solarbio (China). Hoechst was purchased from Beyotime Biotechnology (China). RNA Extraction Solution was purchased from Wuhan Servicebio Technology Co., Ltd. Lipofectamine® 3000 Transfection Reagent (Invitrogen, CA, USA) and Opti-MEM (Reduced Serum Media) was purchased from Thermos Scientific (USA). Confocal laser scanning microscope (CLSM) images were performed on an Olympus FV3000 CLSM (Olympus, Japan). Frozen tissue sections were prepared byLeica CM1860 UV (Germany). qRT-PCR analysis was completed by Wuhan Servicebio Technology Co., Ltd. Oligonucleotide concentrations were measured on a Thermo Scientific NanoDrop™ UV-Vis Spectrophotometer. All the interferential reagents were prepared based on published literature. Mice were purchased from Liaoning changsheng biotechnology (China).

Fluorescence Turn-On and Turnover Measurements.

A 96-well assay plate (black plate and clear-bottomed) was used for all in vitro experiments, and fluorescence measurements were made using a Varioskan LUX multimode microplate spectrometer. Each well contained 100 μL of RNA-**Cy3Tz** and RNA-**ABN** (1 μM) in PBS. The fluorescence emission signals were reported as an average of values from three replicates.

Calculation of the Detection Limit.

The fluorescence intensity of **RNA-Cy3Tz** and **RNA-ABN** or **RNA-BCN** was measured three times, and the standard deviation of the blank measurement was achieved. The detection limit was calculated with the following equation: Detection limit = 3*σ* / *k*, where *σ* is the standard deviation of the blank measurement, *σ*_ABN_ = 0.00124 and *k* is the slope of the fluorescence intensity versus miRNA template concentration growth (*k*_ABN_ = 0.0067).

Calculation of the Catalytic Turnover Numbers (TON)

To quantitatively evaluate the catalytic amplification efficiency of the **IDCR** system, we performed kinetic analysis based on the turnover numbers observed at varying target concentrations. The catalytic turnover number (TON) was calculated using the equation: Turnover Number (TON) = (*F*_target_ - *F*_0_) / (*F*_max_ - *F*_0_) ×1000 nM / [Target], where TON represents the number of catalytic cycles per target molecule in the reaction time (120 minutes).

Cell Incubation

4T1 (mouse breast cancer) cells, MCF-7 (human breast adenocarcinoma) cells, PANC-1 (human pancreatic cancer) cells, HepG2 (human hepatoma cell lines) cells, and NIH-3T3 (mouse embryonic fibroblast) cells was purchased from the Institute of Basic Medical Science (IBMS) of the Chinese Academy of Medical Sciences and cultured with Dulbecco's modified Eagle's medium (DMEM, Invitrogen) supplemented with 10% fetal bovine serum (FBS) and 1% penicillin/streptomycin and seeded in 25 cm^2^ culture flask at 37 °C under a mixture of 5% CO_2_ and 95% humidity. The cell viability was measured by the reduction of 3-(4,5-dimethyl-2-thiazolyl)-2,5- diphenyl-2-H-tetrazolium bromide (MTT). MCF-7 cells were seeded in 96-well microplates at a density of 1 × 10^5^ cells per mL in 100 mL of medium containing 10% FBS buffer. For testing the cytotoxicity of **IDCR**-probe, the cells were cultured in medium with 0.6, 1.2, 2.5, 5, 10, 20, and 30 μM of RNA-**Cy3Tz** and RNA-**ABN** probes for 24 h, respectively. Cells in culture medium without **IDCR**-probe were used as the control (Figure S11). Six replicate wells were used for each control and test concentration.

Live-Cell Detection of IDCR Probe

For imaging experiments, the cells were seeded into a glass bottom dish (35.0 mm dish with 20.0 mm bottom well) with 2.0 mL of medium and cultured in the incubator until the cell density was approximately 80%. To transfect the cells, we incubated 7.5 μL of Lipofectamine 3000 in 125 μL of Opti-MEM in one centrifuge tube (tube A) and incubated 2 μg of RNA-**Cy3Tz** or 2 μg of RNA-**ABN** probes (1:1) in 125 μL of Opti-MEM in another centrifuge tube (tube B). No RNA-**Cy3Tz** probe was added for the negative control reaction. Then, tube B was added into tube A and the solution was mixed until homogeneous. We incubated the probes for 5 min at RT to allow the Lipofectamine-oligo complex to form. Next, the cell media was aspirated from the cells and replaced with 500 μL of the newly made Opti-MEM solution with the complex and incubated for 4 h at 37 °C at 5% CO_2_ and 95% humidity. The cells were washed 2 times using PBS. Fluorescence imaging was performed using an Olympus FV-1000 inverted fluorescence microscope with a 60× objective lens. For microscopy, under the confocal fluorescence microscope, the probe was excited at 488 nm, and emission was collected at 550-650 nm. All of the parameters remained the same throughout the cell experiments.

Fluorescence Imaging of IDCR-probe in Mice Tissue Slices

All animal operations were in accordance with institutional animal use and care9 regulations approved by the Model Animal Research Center of Dalian Medical University (MARC). The animal protocol was reviewed and approved by the local research ethics review board of the Animal Ethics Committee of Dalian University of Technology (approval number: DUTSCE240305-08). All the animal experiments complied with relevant guidelines of the Chinese government and regulations for the care and use of experimental animals. Fluorescence imaging of IDCR-probe in lung slices and subcutaneous lung cancer model was carried out as follows. Lung and subcutaneous lung cancer slices were surgically exposed in BALB/c mice, as approved by the Dalian Medical University Animal Care and Use Committee. The tissues were incubated with 2 μg of RNA-**Cy3Tz** and 2 μg of RNA-**ABN** for 30 min. Before imaging, the tissues were washed with PBS 3 times. An Olympus FV3000 confocal microscope with a 10× objective lens was used for fluorescence imaging. All of these experiments were carried out in accordance with the relevant laws and guidelines.

Analysis of Average Fluorescence Intensity

The images were postprocessed with Olympus FV10-ASW3.0 viewer software. To improve the statistical accuracy, the average fluorescence intensity in cells can be determined from the quantitative analysis of the regions of interest (ROIs) at different locations.

Plasma Separation, Storage, and RNA Collection

A total of 24 mice serum samples were collected. Whole blood was collected in EDTA tubes and processed immediately or within 2 h after storage at 4°C to separate plasma and cellular components by centrifugation at 1900 × g for 10 min at 4°C. The upper (yellow) plasma phase was carefully transferred to a new tube (with conical bottom) and centrifuged a second time at 18000 × g at room temperature to remove any remaining cellular debris and stored at -80°C until the time of RNA extraction.

Fluorescent Assay of Serum Samples.

Human participant experiments were performed with the approval of Medical Ethics Committee of Liaoning Provincial Cancer Hospital (approval number: BQ20241205) and after obtaining informed consent from volunteers. Before detection by probes, each sample was centrifuged under -4°C at 3500 rpm for 10 min for getting rid of the interferents such as grease and red blood cell fragments. A 96-well assay plate (black plate and clear-bottomed) was used for fluorescence measurements. Concentrations of RNA-**Cy3Tz** and RNA-**ABN** were fixed at 1 μM, and the plate wasincubated for 2 h at 37°C. The intensity of fluorescence emission signal was reported as an average of values from three replicates.

RNA extraction experiment

RNA Extraction Experiment. RNA extracts from 4T1, LO2, A549, MCF-7 and PANC-1 cells were prepared using Trizol Reagent according the manufacturer’s protocol. In brief, cells were pelleted at 2000 rpm, 5 min, washed with PBS, and lysed in Trizol Reagent. Subsequently samples were loaded on a column, chloroform added, and the cells pelleted at 10000 × g for 15 min. The water phase was added to isopropanol, and the cells were pelleted at 10000 × g for 10 min. The precipitate was washed with 75% ethyl alcohol and eluted with 200 μL of RNase free-water. RNA concentration was determined using a NanoDrop spectrophotometer to measure O.D. (260 nm), O.D. (260 nm) / O.D. (280 nm) and O.D. (260 nm) / O.D (230 nm) for concentration and purity analysis (Table S1).

Synthesis of Cy3Tz and ABN

Known compound 4, 7, 10, **ABN** was synthesized according to literature.^1,2,3^

***Synthesis of*** ***Cy3-I***

Following a previously developed procedure,^4^ compound 4 (584.85 mg, 1.63 mmol) and compound 7 (500 mg, 1.63 mmol) were dissolved in pyridine (2 mL), then triethyl orthoformate (242 mg, 1.63 mmol) was added. After the mixture was stirred at 100℃ for 3 h, and the reaction progress was monitored by TLC. When the reaction was complete, product was purified by reversed phase chromatography to give pure Cy3-I (298 mg, 27%) as a unsymmetrical pink solid in retention time of 21-22 min. ^1^H NMR (400 MHz, MeOD) δ 8.55 (t, J = 13.4 Hz, 1H), 7.96 (d, J = 1.4 Hz, 1H), 7.93 (dd, J = 8.3, 1.6 Hz, 1H), 7.90 (d, J = 1.6 Hz, 1H), 7.77 (dd, J = 8.4, 1.6 Hz, 1H), 7.43 (d, J = 8.4 Hz, 1H), 7.22 (d, J = 8.4 Hz, 1H), 6.54 (t, J = 12.9 Hz, 2H), 4.42 (t, J = 7.1 Hz, 2H), 4.24 (q, J = 7.2 Hz, 2H), 2.84 (t, J = 7.1 Hz, 2H), 1.78 (s, 6H), 1.76 (s, 6H). MS: m/z calcd. for C_28_H_30_IN_2_O_5_S^-^ 633.09, found 633.06.

***Synthesis of Cy3Tz***

To a 30 mL microwave reaction tube, Pd_2_(dba)_3_ (4.62mg, 0.005 mmol) and ligands 1,2,3,4,5-pentaphenyl-1’-(di-tert-butylphosphino) ferrocene (14.34mg 0.020 mmol), tetrazine **10** (66 mg, 0.303 mmol), **Cy3-I** (66 mg, 0.104 mmol), and N, N-dicyclohexylmethylamine (98.39 mg, 0.5 mmol) were dissolved in anhydrous DMF (3.5 mL). The reaction was protected with N_2_ gas and then heated by microwave irradiation (50 °C, 60 min). The reaction solution was cooled to room temperature and product was purified by reversed phase chromatography to afford pure **Cy3Tz** (39 mg, yield 60%) as a purple solid. ^1^H NMR (600 MHz, MeOD) δ 8.60 (t, J = 13.4 Hz, 1H), 8.35 (d, J = 16.2 Hz, 1H), 8.05 (d, J = 1.4 Hz, 1H), 7.99 – 7.91 (m, 2H), 7.82 (dd, J = 8.3, 1.4 Hz, 1H), 7.61 (d, J = 16.2 Hz, 1H), 7.46 (dd, J = 19.0, 8.3 Hz, 2H), 6.56 (dd, J = 13.4, 10.7 Hz, 2H), 4.47 (t, J = 7.1 Hz, 2H), 4.26 (q, J = 7.2 Hz, 2H), 3.00 (s, 3H), 2.88 (s, 2H), 1.84 (s, 6H), 1.81 (s, 6H), 1.44 (t, J = 7.3 Hz, 3H). ^13^C NMR (126 MHz, Chloroform-d) δ 180.06, 178.34, 177.07, 171.18, 169.55, 151.62, 148.50, 146.54, 146.43, 145.76, 137.18, 131.56, 126.73, 125.39, 125.24, 125.15, 116.92, 116.19, 109.30, 108.39, 54.52, 53.72, 36.56, 32.79, 32.41, 26.04, 17.60. MS: m/z calcd. for C_33_H_35_N_6_O_5_S^-^ ＝ 627.24, found 627.25.

Modification and Sequences of IDCR-probe

**Cy3-Tz**, **ABN** and **BCN**, linked to amine-modified oligonucleotide sequences to form RNA-**Cy3Tz**, RNA-**ABN** and RNA-**BCN** was synthesized by Takara Biomedical Technology (Kusatsu, Japan) Co., Ltd. (Dalian, PR China) and purified using high-performance liquid chromatography. The sequences were as follows:

RNA-**Cy3Tz**: 5’-Cy3Tz-mUmGmAmUmAmAmGmCmUmA-3’;

RNA-**ABN**: 5’-mUmCmAmAmCmAmUmCmAmGmU-ABN-3’;

RNA-**BCN**: 5’-UCAACAUCAGU-BCN-3’;

miR-21: 5’-UAGCUUAUCAGACUGA**U**GUUGA-3’;

miR-21-A: 5’-UAGCUUAUCAGACUGA**G**GUUGA-3’.

**
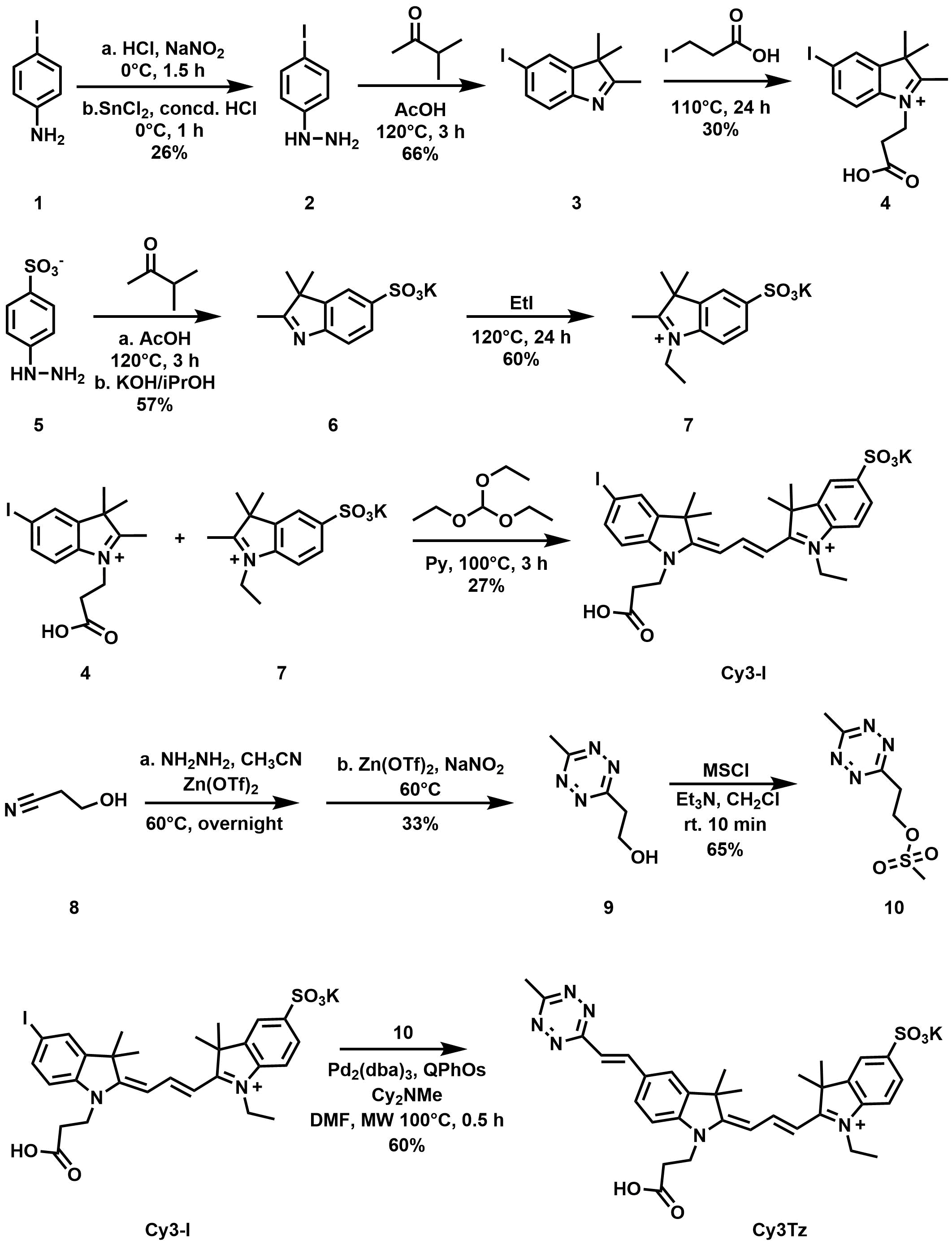
**

**Figure S1**. Synthetic route of **Cy3Tz**.


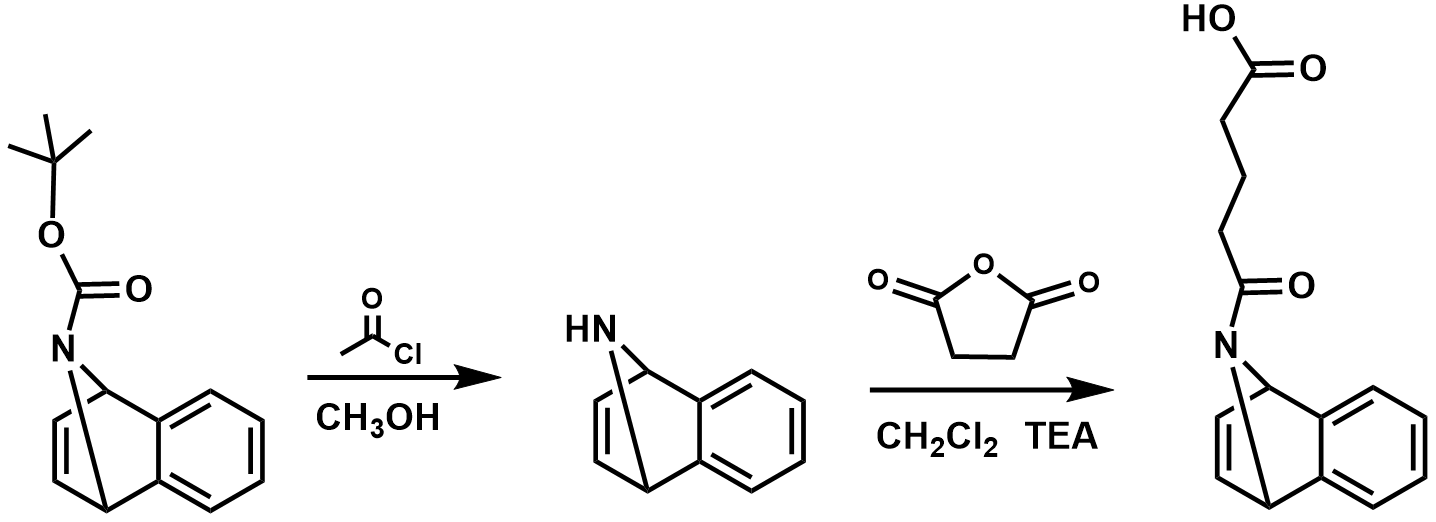


**ABN**

**Figure S2.** Synthetic route of **ABN**.


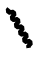

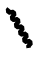

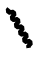

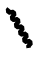

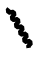

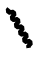

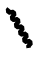

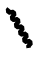


**Figure S3.** Reaction mechanism for Inverse Diels-Alder cyclo-addition reduction

.

**
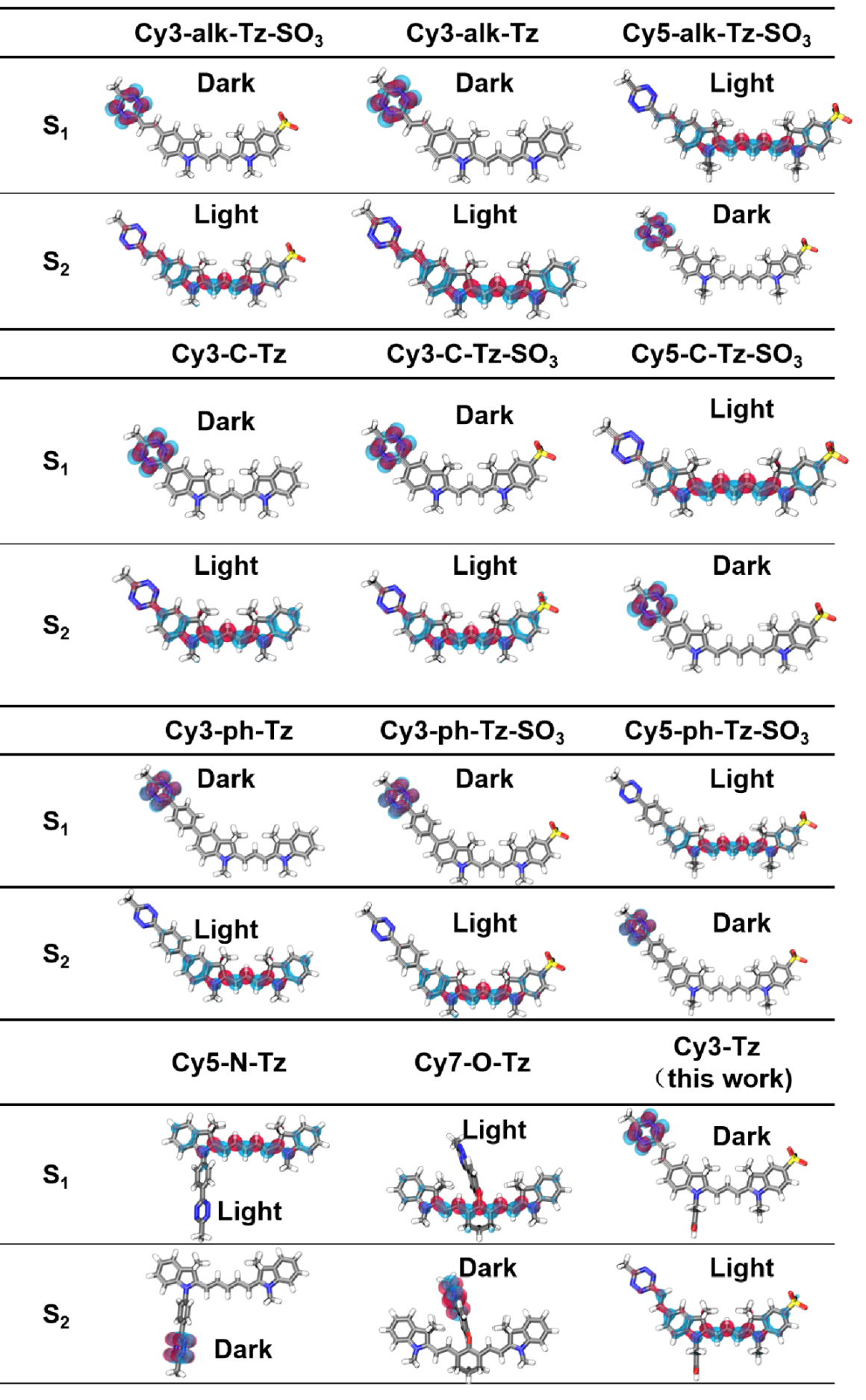
**

**Figure S4**. Optimized geometries, and the corresponding electron, hole distributions of different state of cyanine dyes. (ωB97X-D/Def2-SVP, water as solvent).


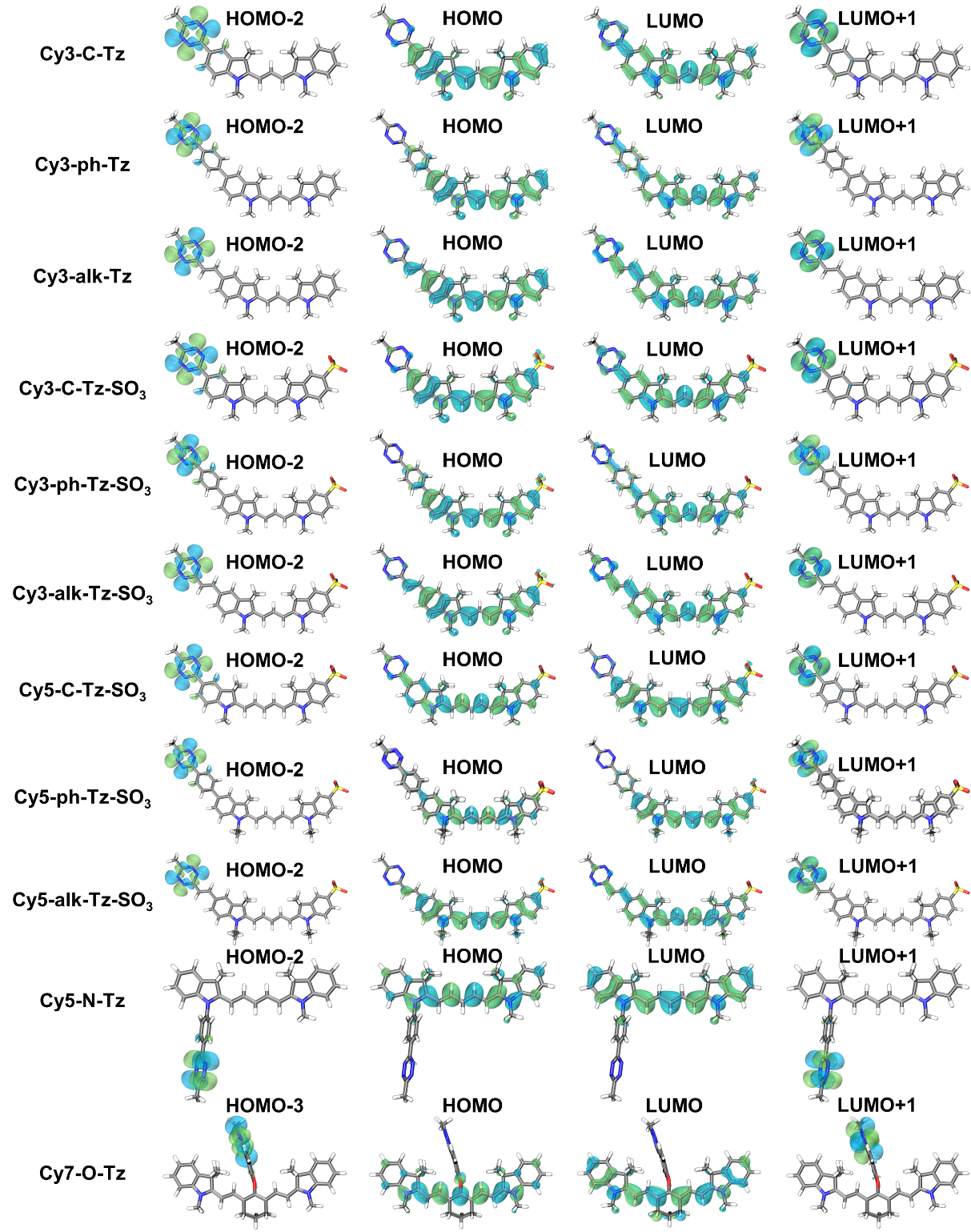


**Figure S5**：Orbital electron distribution of vertically excited states of probes. (M062X/def2-SVP, water as solvent).

Table S1: Orbital energy levels and overlap parameters of vertically excited states of probes


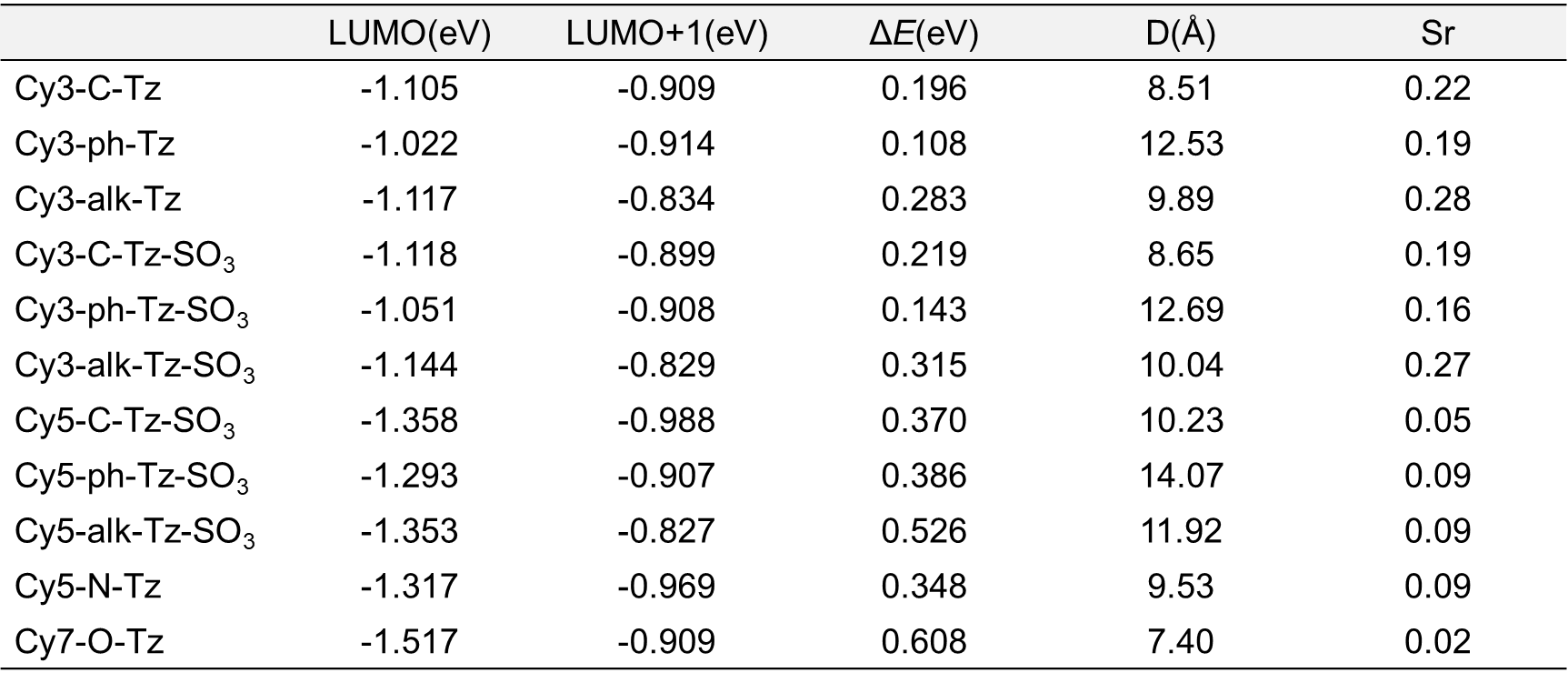


Table S2：Orbital energy levels and overlap parameters of vertically excited states of probes


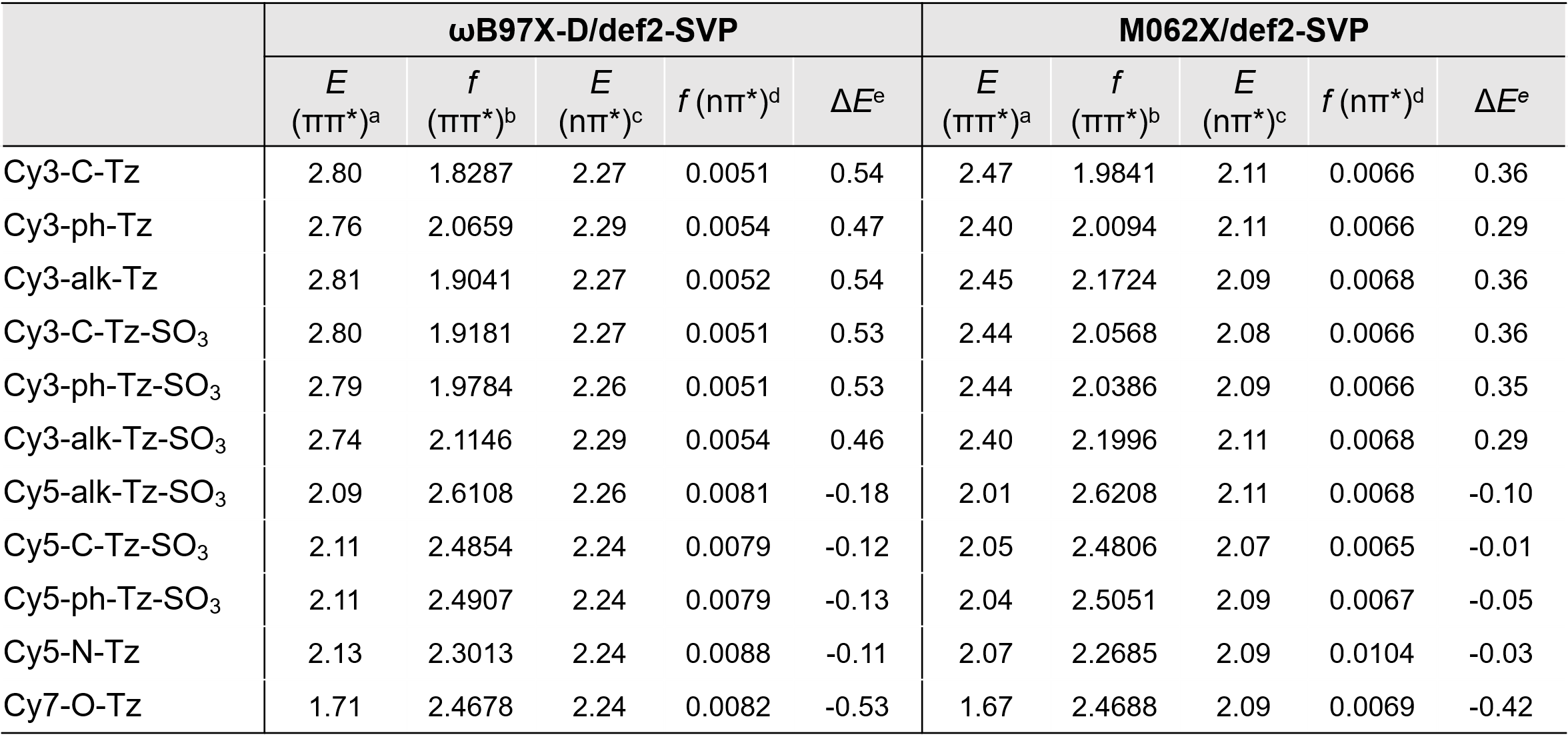


Note: ^a^ Vertical excitation energy of ππ* state (bright state), eV;

^b^ Oscillator strength of ππ* state (bright state);

^c^ Vertical excitation energy of nπ* state (dark state), eV;

^d^ Oscillator strength of nπ* state (dark state);

^e^ E(ππ*)-E(nπ*), eV.

All calculations were performed with water as the solvent using the SMD solvation model.


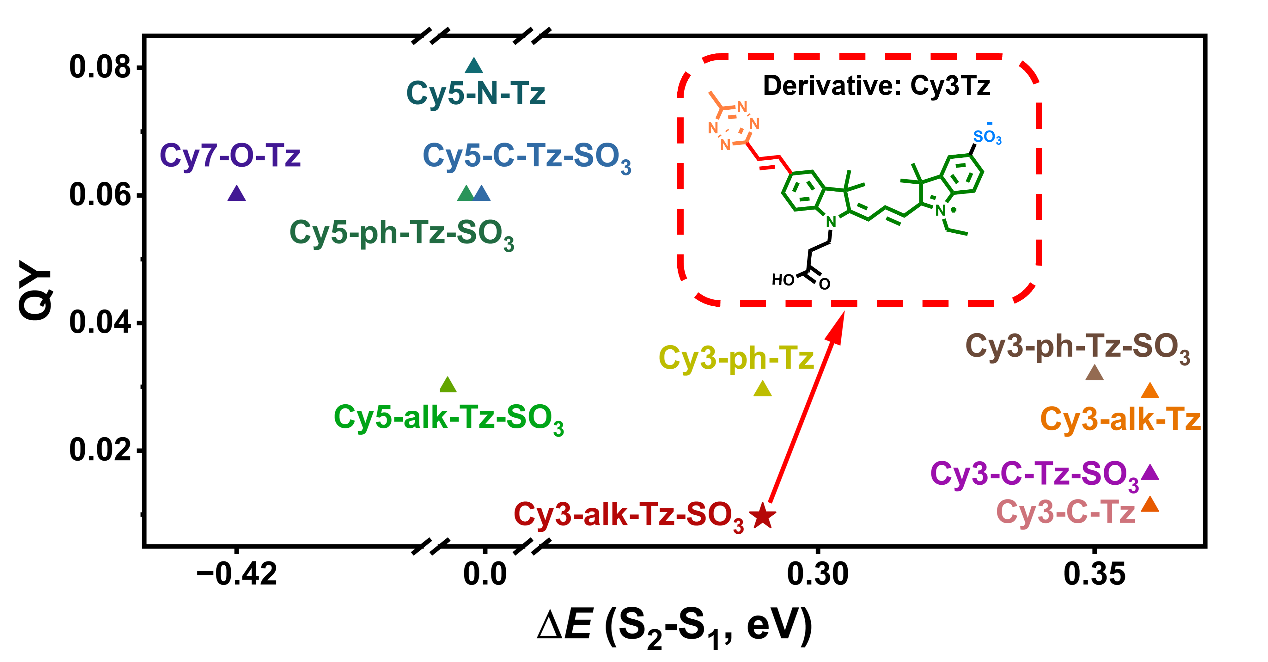


**Figure S6** Correlation of S₂-S₁ gap (Δ*E*) and quantum yield (QY) using M062X/def2-SVP.


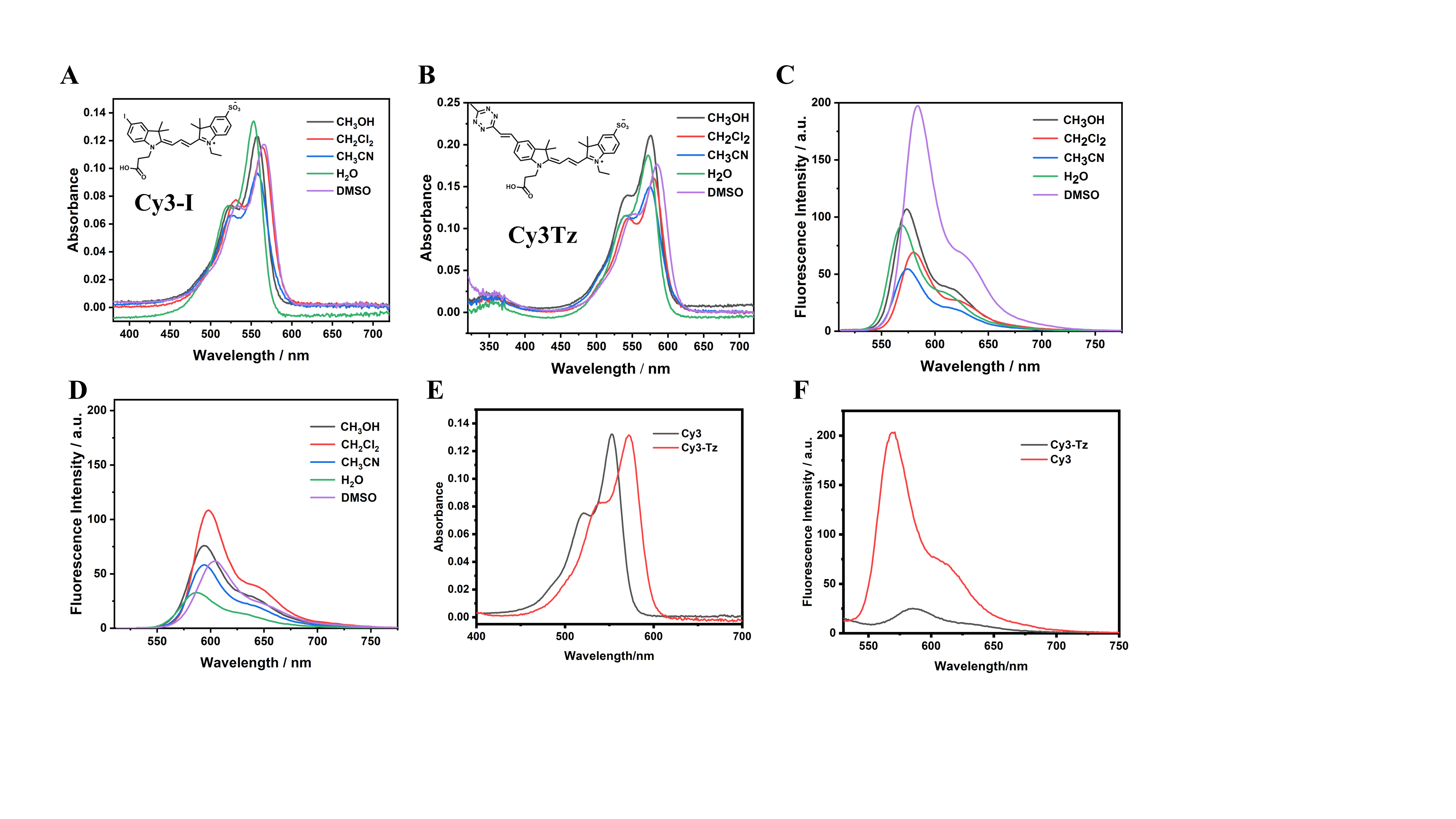


**Figure S7**. The absorption and fluorescence spectra of **Cy3** and **Cy3Tz**.


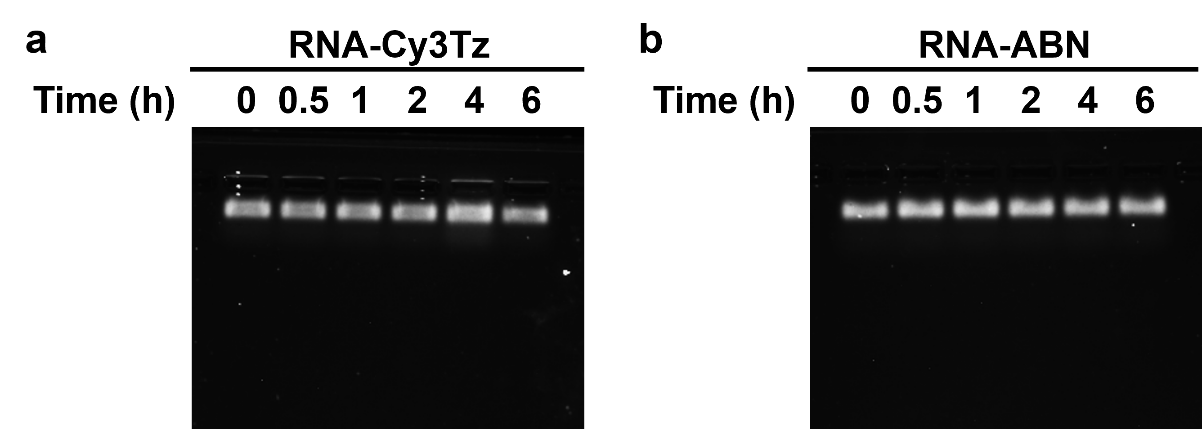


**Figure S8.** Gel electrophoresis analysis of (a) RNA-**Cy3Tz** and (b) RNA-**ABN** after an incubation in DMEM (10% FBS) for indicated time points.

**
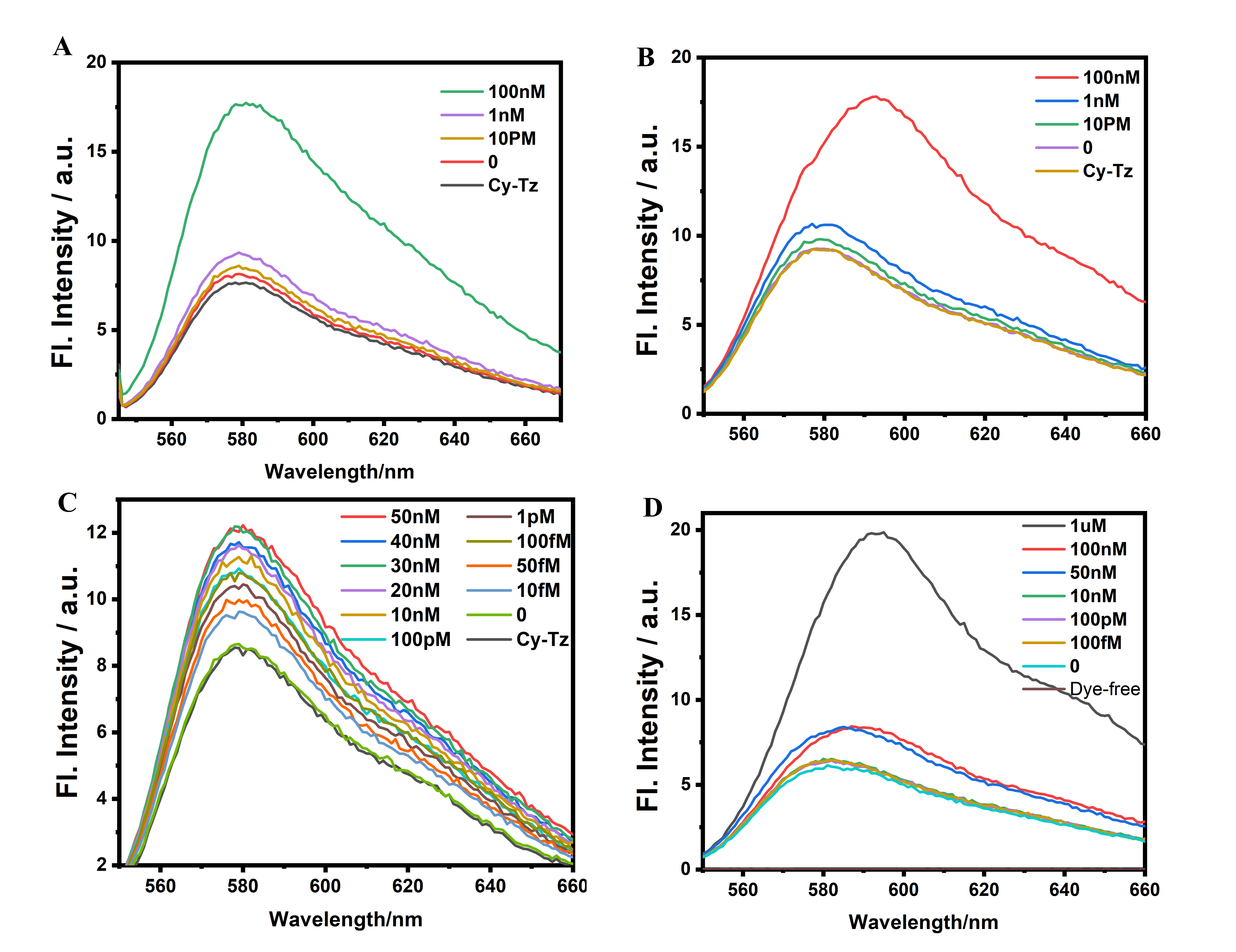
**

**Figure S9**. Feasibility of **IDCR**-probe for miRNA detection in vitro. A) The **IDCR** fluorescence spectrum after 10 minutes of reaction. B) The **IDCR** fluorescence spectrum after 2 hours of reaction. C) Fluorescence spectrum of **IDCR** responding to low concentrations of miR-21. D) Fluorescence spectra of **RNA-Cy3Tz** and **RNA-BCN** in response to miR-21.


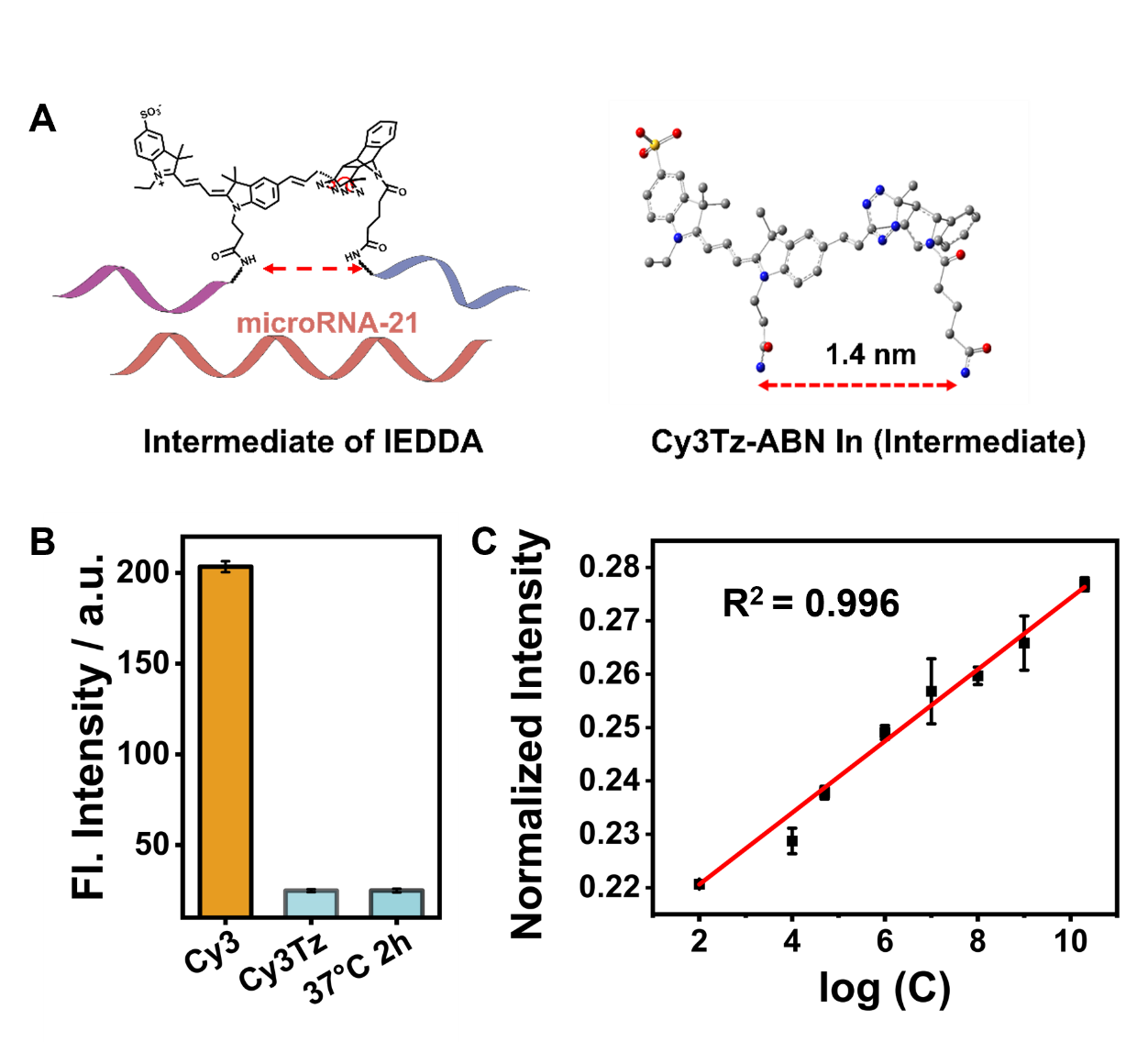


**Figure S10**. (A) Optimized reaction intermediate during the bio-orthogonal reaction between **Cy3Tz** and **ABN**; the red line indicates the distance between the two reactive sites. (B) Fluorescence intensity of Cy3Tz before and after reaction in PBS. (C) Linear relation between normalized fluorescence intensity of the **IDCR**-probe and log(concentrations) of miR-21 template.

**Table. S3 Calculation of Catalytic Turnover Numbers (TON) at different target concentrations.**

| Target Concentration | Fluorescence Intensity (a.u.) | Calculated Product (nM) ^a^ | Turnover Number (TON) ^b^ |
| --- | --- | --- | --- |
| 100 nM | 0.4713 | 336.6 | 3.4 |
| 10 nM | 0.2680 | 81.6 | 8.2 |
| 1 nM | 0.2658 | 78.8 | 78.8 |

Table Notes:

^a^ The calculated product concentration was determined based on the net fluorescence change relative to the blank (*F*_0_ = 0.2030) and the maximum fluorescence of fully reacted probes (*F*max = 1.0000 at 1 μM). Formula: [Product] = (*F*_target_ - *F*_0_) / (*F*_max_ - *F*_0_) ×1000 nM.

^b^ Turnover Number (TON) = [Product] / [Target].

**
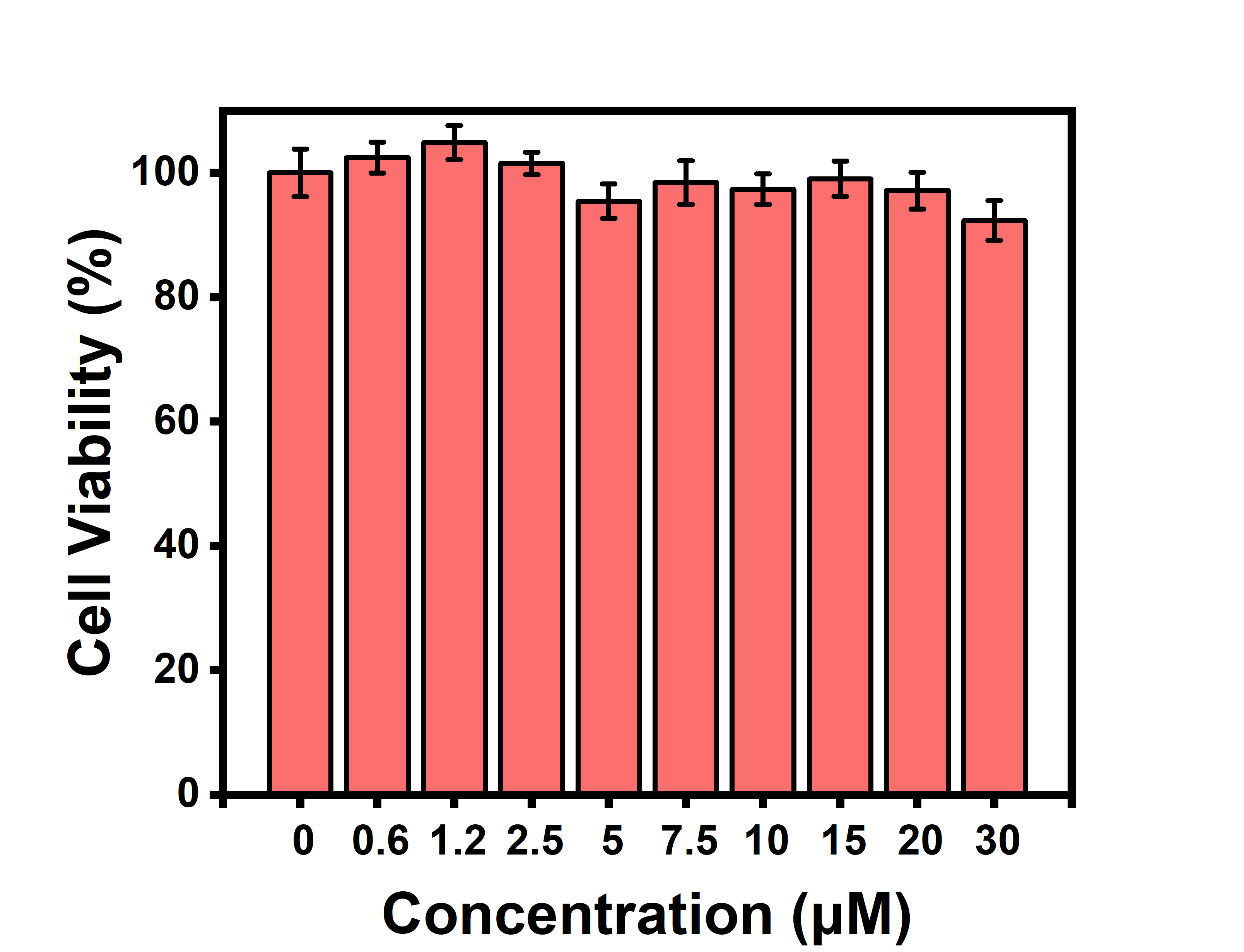
**

**Figure S11.** MTT experiment of MCF-7cells with **Cy3Tz** probe for 24 h.

**
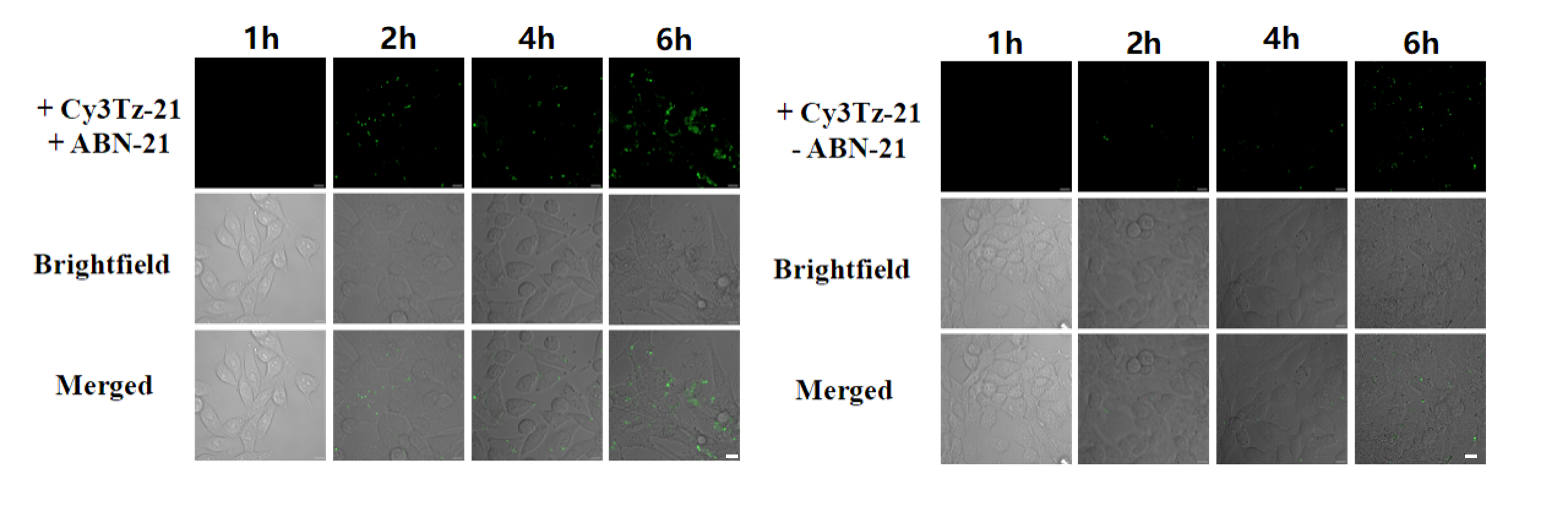
**

**Figure S12.** Endogenous mir-21 imaging in MCF-7 cell with and without + ABN-21 addition. From left to right: RNA-**Cy3Tz** and RNA-**ABN** probes, RNA-**Cy3Tz** probe only. λ_ex_ = 561 nm, λ_em_ = 575 - 675 nm. Scale bar = 20 μm.

**
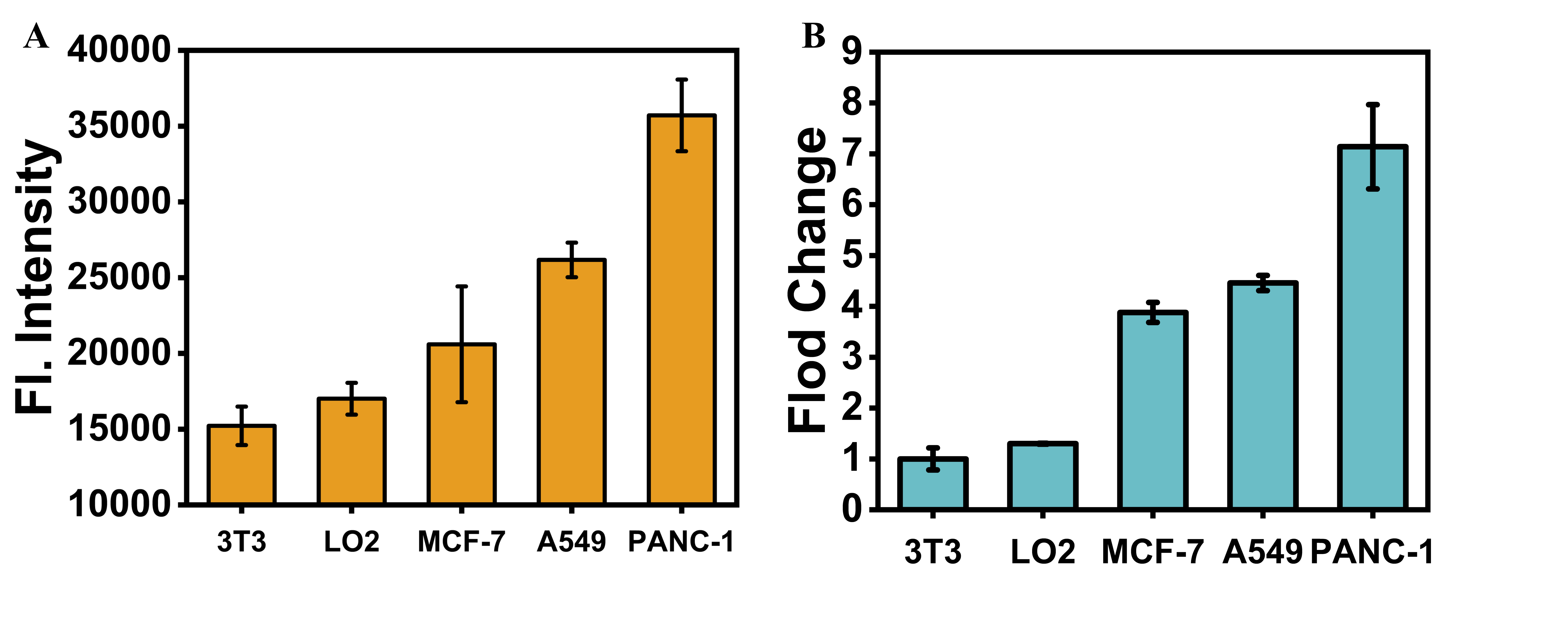
**

**Figure S13.** MicroRNA Detection in biological Samples. Fluorescence responses of endogenous miR-21 in RNA extracts under (A) **IDCR**-probe and (B) qRT-PCR. Error bars show standard deviations of three replicates.

**Table. S4 Comparison of Different miRNA-21 Detection Methods Reported**

| Detection Method | Limit of Detection (LOD) | Signal Readout | Ref. |
| --- | --- | --- | --- |
| **IDCR** Probe | **3.58 aM** | Fluorescence | This work |
| CRISPR/Cas13a + PER | miRNA-21: 2.1 fM  miRNA-375: 4.4 fM | Fluorescence Intensity | *ACS Nano* [1] |
| EXTRA-CRISPR | 1-8 fM | Fluorescence, Smartphone and LFA | *Nature Biomedical Engineering* [2] |
| EXRCA-HCR & AgNPs@gel | 21.47 fM | Fluorescence | *Analytica. Chemica Acta* [3] |
| Palindromic Cascade | 9.7 fM | Fluorescence | *Analytical. Chemistry* [4] |
| Nanopore Sequencing with DNA-Barcoded Probes | ~50 pM | Ionic Current Blockade | *Nature Nanotechnology* [5] |
| Portable POCT (Colorimetric) | 8.3 pM | Colorimetric | *Analytica. Chemica Acta* [6] |

**Table. S5 The quantity of RNA in different cells**

**
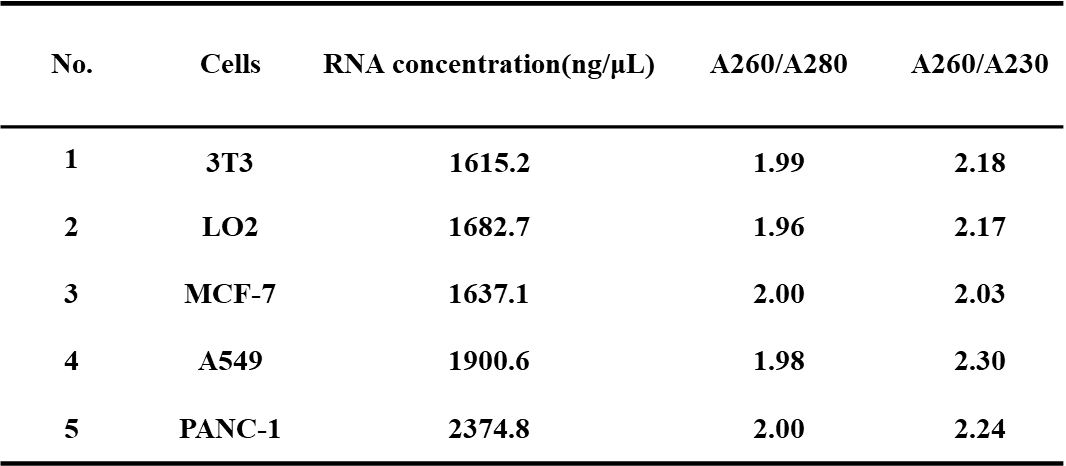
**

**Figure S14.** MS (ESI) of Cy3-I.


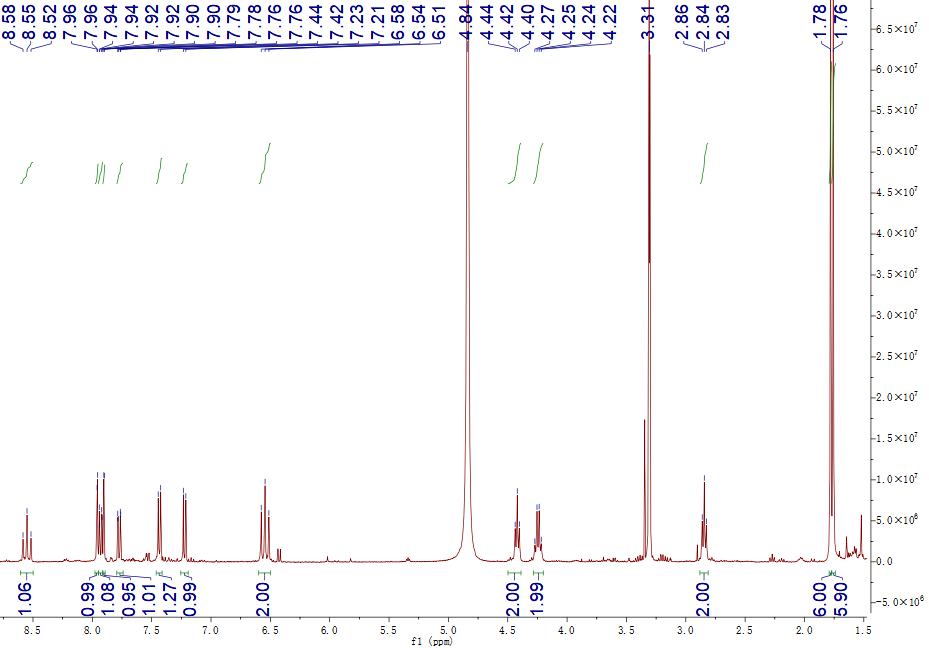


**Figure S15.** ^1^H NMR of compound Cy3-I in MeOD.

**Figure S16.** MS (ESI) of Cy3Tz.

**
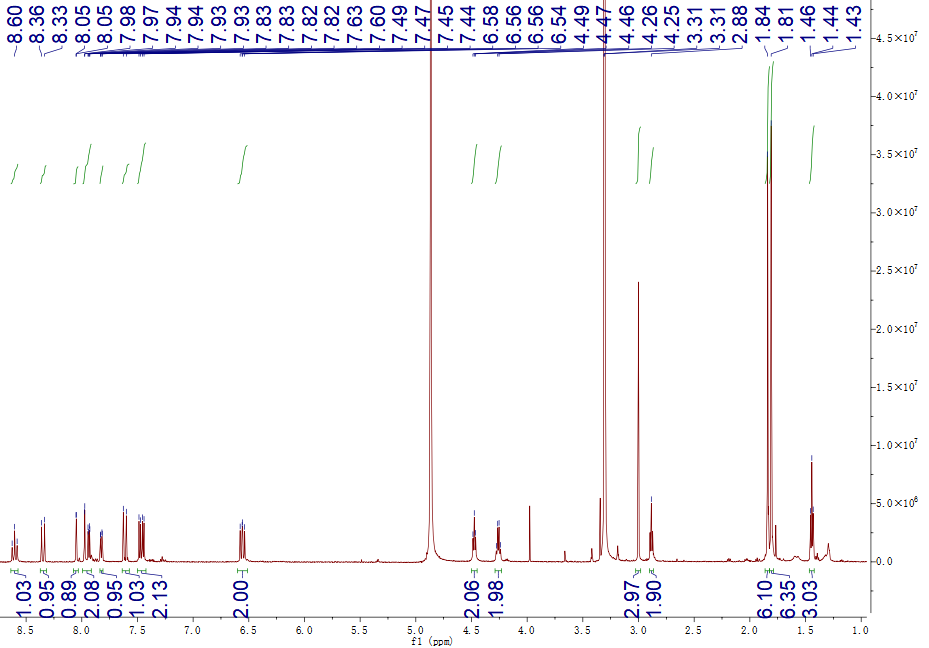
**

**Figure S17.** 1H NMR of compound Cy3Tz in MeOD.

**
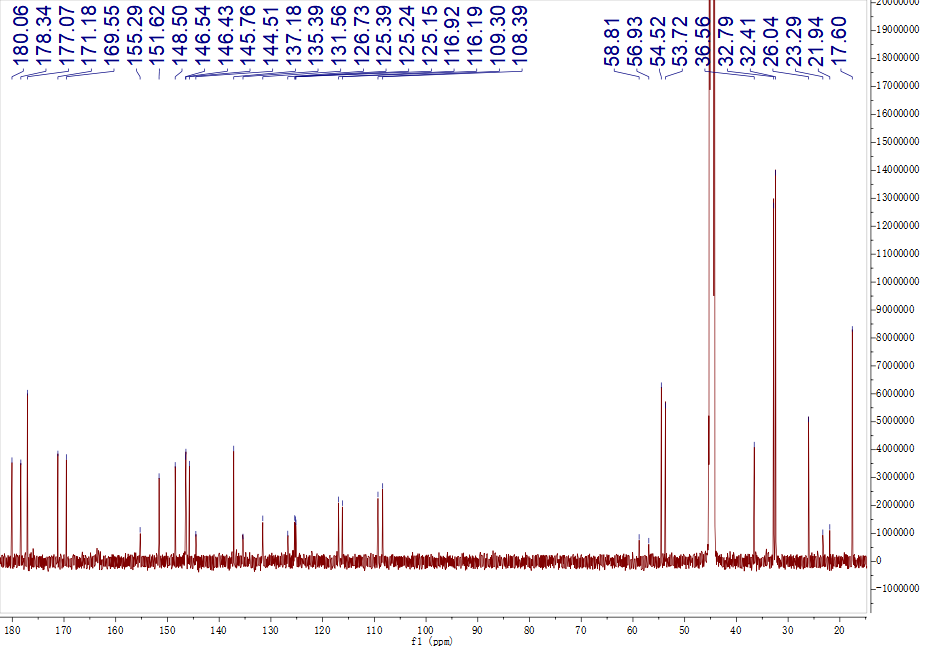
**

**Figure S18.** ^13^C NMR of compound Cy3Tz in MeOD.

**
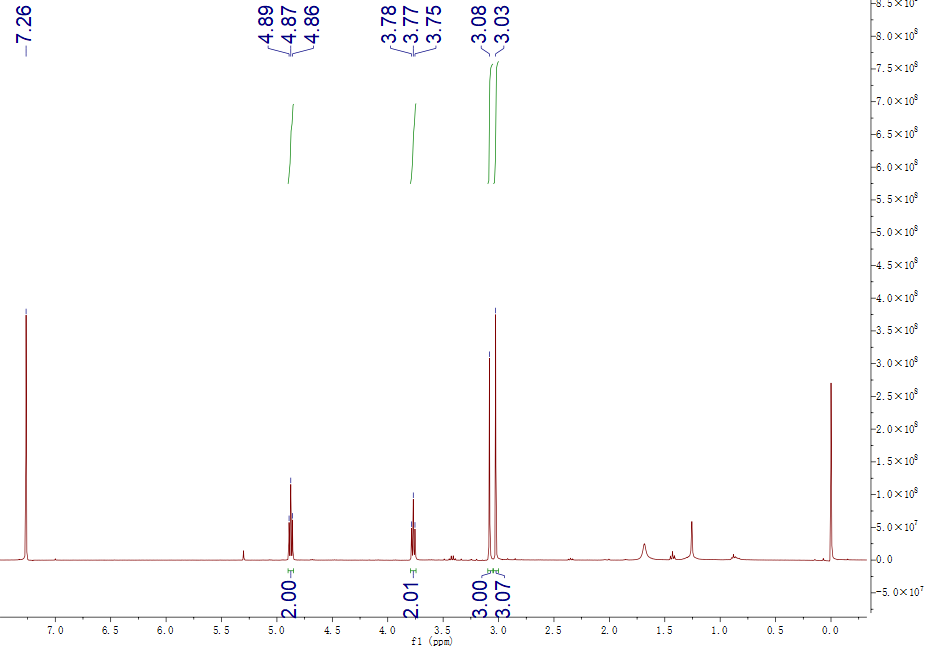
**

**Figure S19.** ^1^H NMR of compound 9 in CDCl_3_.

**
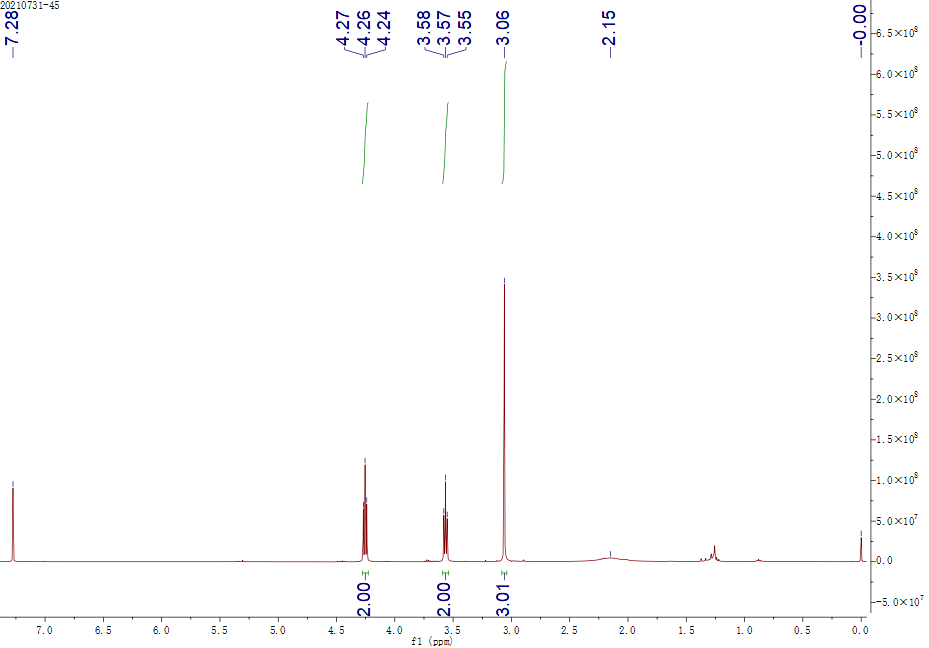
**

**Figure S20.** ^1^H NMR of compound 10 in CDCl_3_.


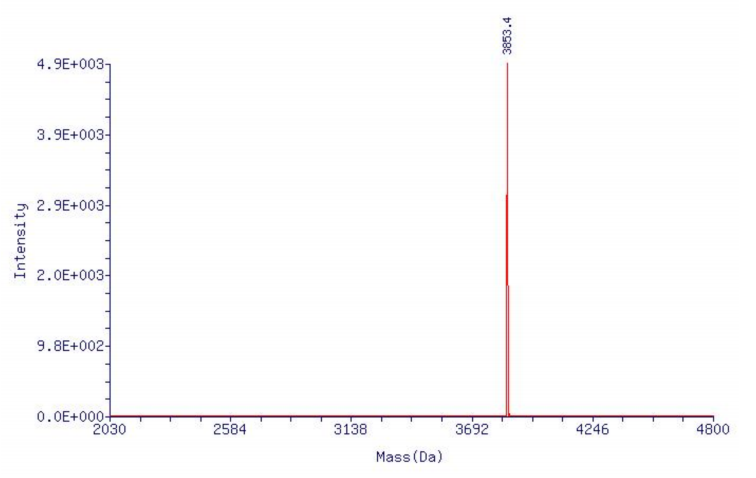


**Figure S21.** MS (ESI) of **RNA-ABN**.


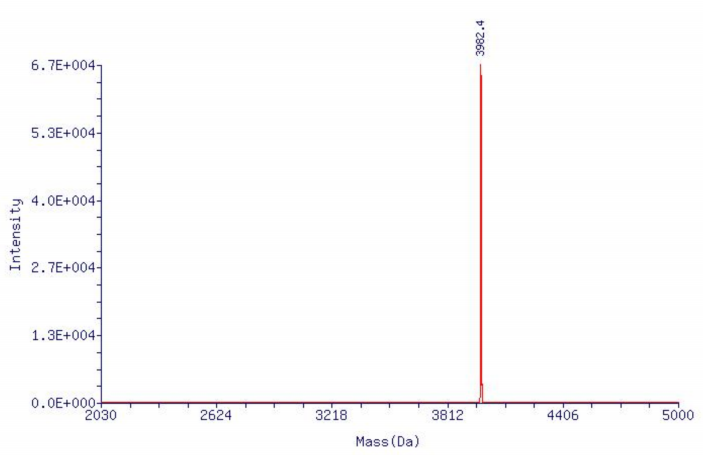


**Figure S22.** MS (ESI) of **RNA-Cy3-Tz**.

# Reference

1. Z. Xie,S. Zhao,R. Deng, et al., "Logic-Measurer: A Multienzyme-Assisted Ultrasensitive Circuit for Logical Detection of Exosomal MicroRNAs" *ACS Nano* **19** (2025): 12222-12236

2. H. Yan,Y. Wen,Z. Tian, et al., "A one-pot isothermal Cas12-based assay for the sensitive detection of microRNAs" *Nature Biomedical Engineering* **7** (2023): 1583-1601

3. W. Zhang,Y. Song,D. Deng, et al., "Exponential rolling circle amplification-hybridization chain reaction (EXRCA-HCR) for AgNPs@gel-enhanced fluorescence ultrasensitive detection of miRNA-21" *Analytica Chimica Acta* **1358** (2025): 344095

4. J. Chen,Y. Shang,Y. Yang, et al., "Palindrome-Mediated Isothermal Cascade DNA Amplification and Effortless Nanosignal Transduction for One-Pot and Ultrasensitive miRNA Sensing" *Analytical Chemistry* **97** (2025): 21012-21020

5. C. Koch,B. Reilly-O’Donnell,R. Gutierrez, et al., "Nanopore sequencing of DNA-barcoded probes for highly multiplexed detection of microRNA, proteins and small biomarkers" *Nature Nanotechnology* **18** (2023): 1483-1491

6. Q. K. Lin,W. Q. Sun,B. Zhang, et al., "A portable point-of-care testing platform for rapid and sensitive miRNA-21 detection for heart failure diagnosis" *Analytica Chimica Acta* **1361** (2025): 344168
